# Antibody characterization using immunosignatures

**DOI:** 10.1371/journal.pone.0229080

**Published:** 2020-03-20

**Authors:** Phillip Stafford, Stephen Albert Johnston, Orhun H. Kantarci, Ameneh Zare-Shahabadi, Arthur Warrington, Moses Rodriguez

**Affiliations:** 1 Department of Bioinformatics, Caris Life Sciences, Phoenix, Arizona, United States of America; 2 Center for Innovations in Medicine, Biodesign Institute, Arizona State University, Tempe, Arizona, United States of America; 3 Department of Neurology, Mayo Clinic, Rochester, Minnesota, United States of America; University of Texas Health Science Center at San Antonio, UNITED STATES

## Abstract

Therapeutic monoclonal antibodies have the potential to work as biological therapeutics. OKT3, Herceptin, Keytruda and others have positively impacted healthcare. Antibodies evolved naturally to provide high specificity and high affinity once mature. These characteristics can make them useful as therapeutics. However, we may be missing characteristics that are not obvious. We present a means of measuring antibodies in an unbiased manner that may highlight therapeutic activity. We propose using a microarray of random peptides to assess antibody properties. We tested twenty-four different commercial antibodies to gain some perspective about how much information can be derived from binding antibodies to random peptide libraries. Some monoclonals preferred to bind shorter peptides, some longer, some preferred motifs closer to the C-term, some nearer the N-term. We tested some antibodies with clinical activity but whose function was blinded to us at the time. We were provided with twenty-one different monoclonal antibodies, thirteen mouse and eight human IgM. These antibodies produced a variety of binding patterns on the random peptide arrays. When unblinded, the antibodies with polyspecific binding were the ones with the greatest therapeutic activity. The protein target to these therapeutic monoclonals is still unknown but using common sequence motifs from the peptides we predicted several human and mouse proteins. The same five highest proteins appeared in both mouse and human lists.

## Introduction

Monoclonal antibodies can bind a variety of targets: lipids, LPS, sugar moieties, phosphorylated or myristoylated residues, conformational or multimeric targets. Therapeutic antibodies often possess characteristics that promote some desired activity *in vivo*. Measurements of affinity can be done using ELISA, SPR or other biochemical assays. However, the human body is a complex environment of proteins, buffers, pH, temperatures, and competitors. Specificity is a measure of off-target binding under very controlled conditions. In the human body, if specificity is high, the antibody might not bind its target *in vivo* due to competition or unsuitable presentation of the target. If too low it might riddle an otherwise effective therapeutic antibody with side effects.

Peptide microarrays have long been used to analyze antibodies against linear epitopes. Tiling epitope arrays can demonstrate the specificity of both polyclonal and monoclonal antibodies [1]. Phage display uses even larger libraries of peptides providing more epitopes to pan, but in both cases the results generally answer the same question: which linear peptide sequences bind to a given monoclonal? A few limitations should be mentioned: antibodies to non-linear epitopes may bind peptides, but they would likely be a mimotope, a sequence unrelated to the sequence of the antigen. Additionally, the peptide arrays likely contain only those peptides needed to cover a single proteome or even just the protein(s) under investigation.

We think it is possible to use a peptide microarray of random sequences to characterize antibodies to both linear and non-linear epitopes. Mimotopes can bind as strong or stronger to an antibody than its original antigen and may exist in a random peptide array of only a few hundred thousand sequences [2]. We have demonstrated that antibodies to linear sequences can find motifs that match their antigen.

When peptides are arrayed on a solid surface, such as a microarray, peptide-antibody interactions can be measured by detecting bound antibody after stringently washing the array. Typically, an antibody is bound strongly when 4–5 residues make a perfect match, generally ~50kCal/mol. Fewer than that and antibodies are washed off. This is how most tiling arrays work; it is undesirable to retain antibodies from imperfect matches. We found that peptides spaced <1nm apart on a solid surface could create a dense forest that enables weakly captured antibodies to be trapped, re-binding to the peptides creating a high local avidity that antibodies with only 2–3 residues need to be a perfect match, being retained even following a stringent wash [3]. This allowed us to see thousands of binding events on arrays with only 125,000 to 330,000 peptides [4].

## Materials and methods

### Training on commercial antibodies

In order to gain understanding of how different antibodies behave on random-peptide arrays, we selected 4 different peptide microarrays that utilize random-sequence peptides, but use different lengths and numbers of peptides (see Table 1). We then purchased 24 different commercial monoclonal antibodies to test on these four peptide microarrays (see Table 2). The epitopes for these antibodies varied substantially, by design. One target is against a hapten, three targets are against proteins in the phosphorylated and dephosphorylated form, eleven are against linear peptides, four of which are <13 residues, and seven are against putative but unmapped regions of proteins. We prioritized our analysis of linear epitopes against the eleven monoclonals to linear peptides.

**Table 1:** Description of peptide libraries used in this project. Four libraries were constructed using mask-based lithography synthesis. Each library is synthesized on silicon wafers coated with silicon oxide. Every library is made in a 0.49cm2 area, 24 separate assays are arrays on a standard 25mm x 75mm slide in an 8x3 design. Every array was assayed under the same conditions.

Library name	# unique peptides	Mean peptide length	SD peptide length	Min, Max	Feature size	Refs
CIM 330K (short)	329239	12.19	1.76	3,17	8um diameter	[9, 14, 16]
CIM 330K (long)	328794	17	0	17	8um diameter	
HT 124K	126051	9.00	1.37	3, 13	14um square	[19]
CIM 125K	122927	12	0	12,12	14 um square	[17, 20, 21]

**Table 2:** Table of commercially-sourced antibodies used. Ab Name is the title of the antibody as sold by the manufacturer. Protein target is the gene name of the protein that served as the antigen. Ab Clone is the nomenclature the manufacturer uses to identify the hybridoma cell lineage. Epitope is the target of the antibody, generally the position in the Protein Target (when known) that contains the exact epitope. Generally, a short Epitope implies a linear peptide was used as the immunogen. aa is the number of amino acids included in the linear immunogen. Source is the manufacturer, along with the catalog number. Host is the mammalian animal host. Last, isotype is the class of antibody produced.

Ab Name	Protein Target	Ab Clone	Epitope	aa	Source	Cat #	Host	Isotype
**AKT1 (B-1)**	huAKT1	B-1	aa345-480	135	Santa Cruz Biotech	sc-5298	Mouse	IgG1
**AKT1 (7)**	huAKT1	7	aa71-184	113	Santa Cruz Biotech	sc-135829	Mouse	IgG1
**AKT2 (D-17)**	huAKT2	Sc-7127	aa445-470	25	Santa Cruz Biotech	sc-7127	Goat	IgG1
**p53 Ab1**	huTP53	PAb240	aa376-380, RHSVV	5	EMD Millipore		Mouse	IgG1
**p53 Ab8**	huTP53	BP53-12	aa20-26, SDLWKL	7	Invitrogen	MA1-19055	Mouse	IgG1
**DM1A**	huα-tubulin	Sc-32293	aa 426–432, AALEKDY	7	Santa Cruz Biotech	sc-32293	Mouse	IgG1
**JNK2**	huJNK2	D-2	aa1-424	424	Santa Cruz Biotech	sc-7345	Mouse	IgG1
hnRNP-A1	huRNA-A1	4B10	aa1-320	320	Novus Biologicals	NBP1-99106	Mouse	IgG1
FDFT1	huFDT1	3092621	N-term FDFT1	N-term	Abgent	BP2417a	Mouse	IgG1
p21	huP21	187	unknown	unknown	Santa Cruz Biotech	E2705	Mouse	IgG1
p-p21	huP21 *P	Ser 146-R	Phos Ser146	unknown	Santa Cruz Biotech	Sc-12902	Mouse	IgG1
p27	huP27	F-8	aa 1–197	197	Santa Cruz Biotech	Sc-1641	Mouse	IgG1
p-p27	huP27 *P	Thr 187-R	Phos Thr187	unknown	Santa Cruz Biotech	Sc-16324-R	Rabbit	IgG
**p38**	huP38	A-12	aa 213–360	143	Santa Cruz Biotech	Sc-7972	Mouse	IgG1
p-p38	huP38 *P	Sc-7973	unknown	unknown	Santa Cruz Biotech	Sc-7973	Mouse	IgG1
p-ERK	huERK1 *P	E-4	Tyr 204	unknown	Santa Cruz Biotech	A0606	Mouse	IgG2a
**Cyclin B1**	huCyclin B1	D-11	aa 1–433	433	Santa Cruz Biotech	L0604	Mouse	IgG1
IFN-y	huIFN-y		unknown	unknown	Pharmingen	551216	Mouse	IgG1
biotin IFN-y	huIFN-y biot	IFN-y	Iml	unknown	Pharmingen	554410	Rat	IgG
6A7 Bax	huBAX	unknown	FL	FL	Pharmingen	556467	Mouse	IgG1
Anti-BrdU	Bromo-uracil		BrU	1	Thermo-Fisher	A23210	Mouse	IgG1
SR 1H4	xlSR	1H4	FL	FL	Santa Cruz Biotech	Sc-13509	Mouse	IgG1
Clk1	huCLK1	G313-1	FL	FL	BD Biosciences	556388	Mouse	IgG1
**LNKB B2**	huIL2	A641	KPLEEVLNL	9	Geneway	GWB-A641EC	Mouse	IgG1

### Testing blinded therapeutic antibodies

Our collaborators at the Mayo Clinic in Rochester, MN supplied us with thirteen different therapeutic antibodies (Table 3). The characteristics of these antibodies were blinded to us other than to state that they were of mouse or human origin and that they were IgM. These antibodies had previously been tested for their ability to remyelinate the central nervous system (CNS) as an approach to remediate symptoms from Multiple Sclerosis[5]. These monoclonals were sourced from Waldenstrom’s myeloma cells, and are IgM rather than IgG[6]. They were selected due to their unusual properties that causes remyelinating activity *in vivo* [7, 8]. Mayo’s experiments revealed that the most efficacious of these antibodies did not halt demyelination, rather it initiated remyelination of neurons during periods of remission. This effect could potentially be leveraged to restore function to humans recovering from MS. Five mouse and eight human antibodies were selected, deidentified, and sent to Arizona State University for processing on the 330,000 peptide immunosignature array.

**Table 3:** List of therapeutic antibodies from Mayo. These antibodies were shipped from Mayo (Rochester, MI) to ASU (Phoenix, AZ) blinded, labeled only by the source (human or mouse) and the number (Antibody ID). The Code, Specificity in CNS and the Function were known only to Mayo prior to unblinding. Human IgM-6 (Code 22) is in human trials for remyelination. Mouse IgM-5 (Code 94.03) was shown to promote remyelination, and was identified as a natural autoantibody.

Antibody DeID #	Source	Code	Specificity in CNS	Function
Hu IgM-1	Human	201	Neurons	Neuronal Extension
Hu IgM-2	Human	236	Neurons	Neuronal Extension
Hu IgM-3	Human	242	Neurons	Neuronal Extension
Hu IgM-4	Human	248	Neuron	Neuronal Extension
Hu IgM-5	Human	263	Neurons	Neuronal Extension
Hu IgM-6	Human	22*	Oligodendrocyte	Remyelination
Hu IgM-7	Human	42	Oligodendrocyte	Remyelination
Hu IgM-8	Human	297	Neuron	Neuronal Extension
Mm IgM-1	Mouse	A2B5	Progenitor	Remyelination
Mm IgM-2	Mouse	O4	Oligodendrocyte	Remyelination
Mm IgM-3	Mouse	O9	Oligodendrocyte	Remyelination
Mm IgM-4	Mouse	79.08	Oligodendrocyte	Remyelination
Mm IgM-5	Mouse	94.03**	Oligodendrocyte	Remyelination

We followed the same procedure used for the commercial monoclonals: exploratory data analysis of the data distributions, general patterns and commonality among the antibodies.

### Peptide synthesis and array printing

Four different random peptide microarray libraries were used (Table 1), two with ~330,000 peptides and two with ~125,000 peptides. Some arrays had peptides shorter or longer length; some libraries had fixed length peptides; some had peptides of variable length. Each library was treated the same relative to sample processing and analysis. Each array is repeated 24 times on one standard slide with a gasket separating the assays. Synthesis of the peptides on the silicon wafers was performed as described [9], using shadow-mask lithography and BOC peptide synthesis. The assay is performed as follows: first, arrays are incubated in the presence of sample buffer (SB = 1x PBS pH 7.3 + 0.05% Tween20 (Sigma-Aldrich, St. Louis. MO) for one hour at 25°C with gentle agitation. Antibodies were added by multichannel pipette to the arrays to a final concentration of 4nM in 150ml of sample buffer. The primary incubation is done at 37°C in a rotating hybridization oven (Agilent, Santa Clara, CA) for 1hr. The gasket is removed, the slide washed 3x in SB for 5 minutes with agitation, then 3x5 minutes each in deionized 50MW water with agitation. The slides are placed in a 5ml tray without the gasket, where 2ml of SB + 5mg/ml casein (Fisher Scientific, Fair Hills, WI) at pH 7.3 is added and fluorescent anti-mouse (Jackson Lab AlexaFluor555 goat anti-mouse IgM Fc) or anti-human (Life Technologies AlexaFluor555 mouse anti-human Fc) secondary antibody is added to a final concentration of 4nM. The secondary binds to the primary antibodies for 1hr at 25°C with gentle agitation. Slides are washed as above, dried by centrifugation at 1500g for 10 minutes, then scanned at 1um resolution in an Innopsys Innoscan 910 two-channel scanner at high laser power, 20% PMT. A 16-bit TIFF image is stored for each array (24 images per slide), aligned using GenePix Pro 6.0, data analyzed using GeneSpring 7.3.1 (Agilent, Santa Clara, CA) or R (CRAN Repository).

### Analysis methods

We first asked whether there were any generalizable measures of binding that could differentiate the 24 antibodies. We first analyzed the data distributions for trends or patterns using EDA (exploratory data analysis) across all four different peptide libraries. We then asked whether there were differences in these trends on libraries with shorter or longer peptides (330K long and 330K short libraries), or on arrays with fixed length (HT124K) vs. variable length (CIM125K). We compared Shannon’s entropy, data distribution, binding promiscuity, and dynamic range. Finally, we asked whether we could find evidence of the eliciting linear epitope for those monoclonals raised to them in the short, random peptides. Once we competed these preliminary experiments, we tested a collection of antibodies blinded to us provided by a collaborator at Mayo, Rochester.

To capture sequence information from the peptides per monoclonal, we analyzed the top 200 peptides that uniquely bound to each monoclonal. These peptides and their binding intensities are shown in the heatmaps in Fig 4. The test to identify peptides specific to each monoclonal used a correlation score that compared a vector that simulates a pattern representing the highest signal for that monoclonal but the lowest possible signal for every other monoclonal. Any peptide that matched this hypothetical pattern produced a high correlation and appeared at the top of the list. This list produced peptides that bind strongly to just one monoclonal. We used CLUSTALW (GNU General Public License, v2) to group the peptide sequences into clusters, and asked GLAM2 [10] to align the peptides from CLUSTALW using gaps (if necessary) to find the most conserved positions in a motif. The resulting motifs were searched for the peptide when known. This was done for 10 of the monoclonals (bold/underlined in Table 2).

## Results

### Immunosignature training on 24 monoclonals

The peptide arrays produce binding data between antibody and peptides. This pool of data is called an immunosignature. An immunosignature is compilation of the steady-state binding affinities between the library peptides and the antibody (or collection of antibodies, as found in serum). Immunosignatures are generally log_10_ normal [3, 11]. However, a single antibody can have a broad range of binding characteristics and the binding data may deviate from log_10_ normality. This measure of the data distribution can be considered the first of many general observations. As in epitope mapping experiments, the highest-binding peptide sequences for antibodies raised against linear peptides can be measured, clustered, and examined for motifs that should correspond to the linear epitope for that antibody.

**Fig 1:**
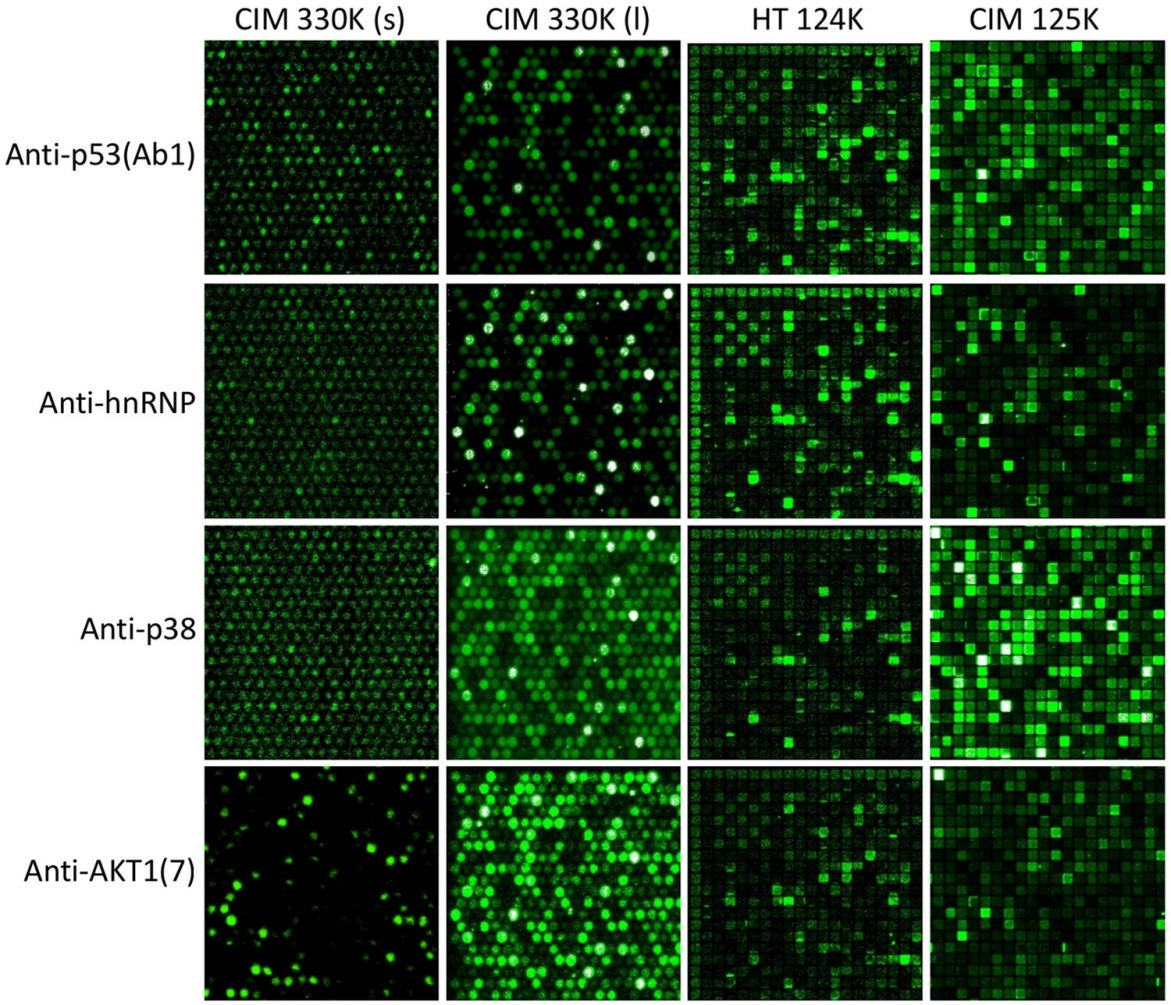
Raw images of a small portion of the upper-left portion of four different peptide microarrays showing four different monoclonal antibodies. The X axis is four different peptide libraries; CIM330K (short) is a library of 330,000 random-sequence peptides of length 12.2 residues; CIM 330K (long) is a library of 330,000 random-sequence peptides of length 17 residues. HT124K is a library of 124,000 random-sequence peptides of length 9 residues; CIM125K is a library of 125,000 peptides of mean length 12 residues. The Y axis is four commercially-sourced monoclonal antibodies. Row 1: anti-human TP-53 (Ab1) has low binding to most peptides but very high binding to a small subset of peptides, especially to those containing the sequence RHSVV. Row 2: anti-human hnRNP monoclonal has an intermediate binding prevalence, with approximately 15% of the total peptides binding at >2SD above background. Row 3: anti-human p38 monoclonal has low binding pattern but at least 15% of all peptides bind at least 2SD above background with a few high binders. Row 4: anti-AKT1(7) monoclonal has more promiscuous binding with >40% of all peptides binding >2SD above background for HT124K and CIM125K. This visual display is intended to demonstrate qualitatively how diverse the binding patterns are.

Fig 1 shows images from the four peptide libraries that were used: the 330K (short) library consisted of peptides of varying lengths, aveage length was 12.2 residues. The 330K (long) library consisted of peptides of length 17 residues. The HT 124K is a commercial peptide array made by HealthTell (San Ramon, CA) with a mean length of 9 residues. The CIM 125K is similar to the HealthTell arrays, with a mean length of 12aa. Previous reports indicate that even short motifs found in an immunosignature peptide can be statistically relevant to the linear epitope of an antibody [12–16] but it was unknown how the size of the library impacted the ability to predict epitopes.

**Fig 2:**
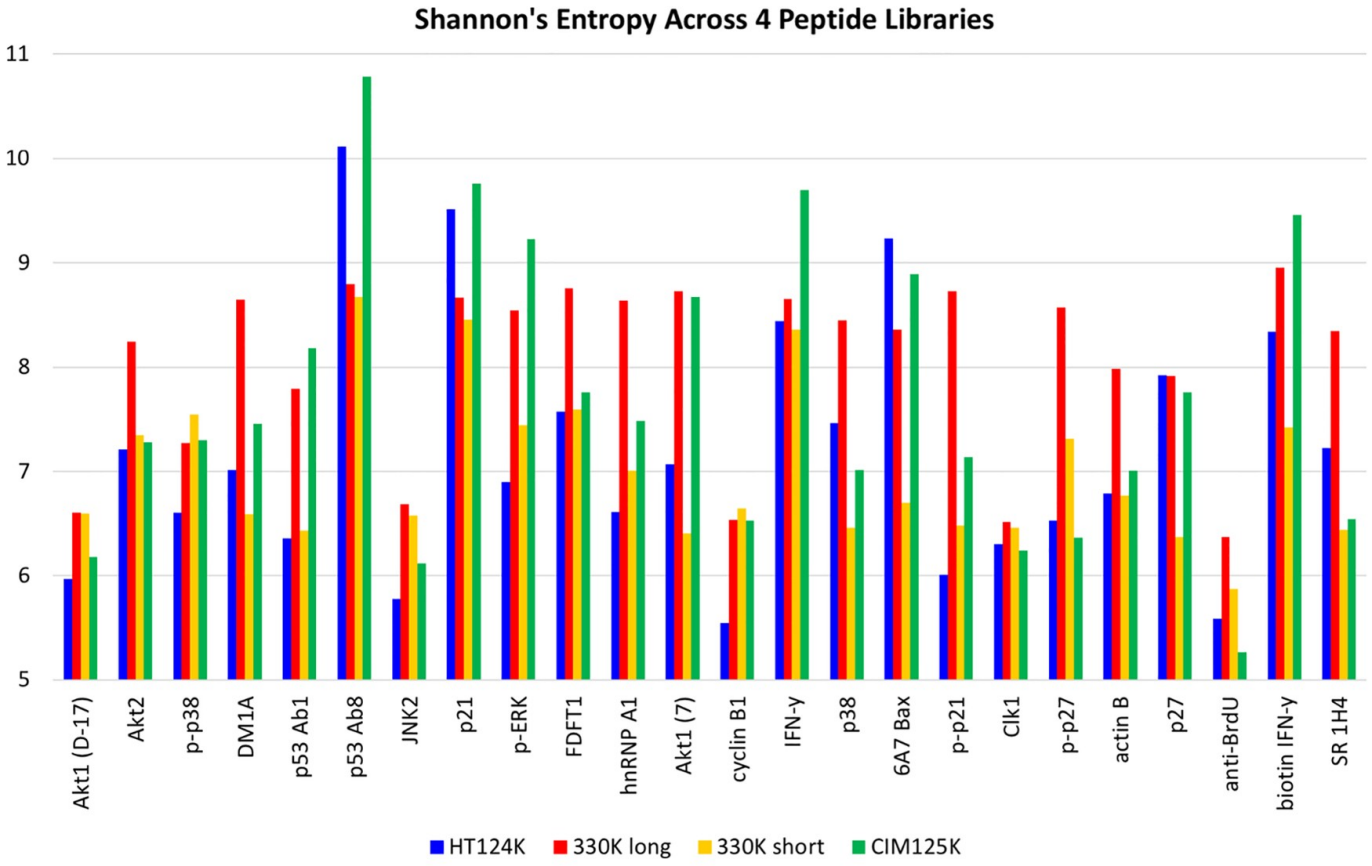
Entropy measures of each of the 24 different monoclonals tested. Shannon’s Entropy was calculated for each of the monoclonals and each of the 3 different peptide libraries. Since each peptide library is different, entropy calculations will differ as well, however a general trend shows that p53Ab8 has generally high measured entropy and anti-BrdU the lowest.

Table 4 and Fig 2 illustrate a method that uses Information Theory to determine whether there is a difference in the diversity or randomness found in the binding pattern. Table 4 lists several descriptors of data distributions (mean, stdev, upper 95^th^ percentile, skewness, kurtosis, dynamic range). We settled upon Shannon’s Entropy as it provided a wide range of values that should indicate diversity of information content. We applied Shannon’s Entropy [17] to the *patterns* of data for each monoclonal for each random peptide library. Entropy is highly dependent on the composition of the library, thus there are larger differences in the score between libraries than between antibodies using the same library. Entropy scores are constrained by each library. Therefore, the rank order of scores for antibodies is the best way to compare each antibody across the different peptide libraries. Entropy was found to be more sensitive to subtle changes in the relationship between the peptide binding pattern and biological associations. For example, the entropy score for an immunosignature increases (more randomness) as additional monoclonals were added to the solution that bound to the microarray. Thus, for a single monoclonal, an entropy score would be high if that monoclonal were more promiscuous, but also a strong binder [17]. The antibody p53 Ab8 had the highest *relative* entropy score across all libraries and has shown strong and very specific binding to very few peptides, generally when at least 2–3 amino acid residues of its cognate epitope are present and only when they contain the tryptophan (SDLWKY). By comparison, anti-BrdU antibody was raised against a small hapten and binds many peptides strongly.

**Table 4:** List of selected characteristics of the data distributions from each of the peptide array formats. The table below lists seven different numerical descriptors of the full dataset from each peptide microarray library (330K short peptide, 330K long peptide, 125K short peptide, 124K short peptide), and for each antibody. In order, the descriptors are: Shannon’s Entropy [17], 95^th^ percentile coefficient of variance (calculated from the standard deviation / mean of the 95^th^ upper percentile of all observed fluorescence intensities for each antibody per peptide library), the mean of all intensities, the stdev of all intensities, the measured kurtosis of all raw intensities, the skewness of the raw intensity data distribution, and the dynamic range of the raw intensities.

**Antibody**	**CIM330K short**	**CIM330K short**	**CIM330K short**	**CIM330K short**	**CIM330K short**	**CIM330K short**	**CIM330K short**
	Entropy	95^th^%ile var	mean	stdev	kurtosis	skewness	DR
AKT1(B-1)	6.59699095	0.94827464	997.931681	663.127174	2854.00735	34.8141055	4.97326203
AKT1(7)	6.40821724	0.43249303	742.91363	603.430384	5633.04555	55.6273088	6.21982759
Akt2(D-17)	6.34880064	0.16847798	872.804152	612.759423	3395.00949	38.431947	5.66894198
p53 Ab1	6.43404063	0.89970233	839.422526	749.430333	3689.28369	47.0647225	6.72727273
p53 Ab8	6.67474666	0.58985062	743.369711	720.764303	3992.68735	50.5967494	5.98739496
DM1A	6.58937783	0.35993306	1000.65025	955.97848	2427.36127	40.1117727	4.96216216
JNK2	6.57656755	0.16700709	960.27086	700.313264	2740.54469	35.8329888	5.55246914
hnRNP-A1	7.00998041	0.77525392	847.922562	548.851338	2770.05958	27.4521631	7.05416667
FDFT1	6.5936509	0.42609586	938.274437	1196.20813	1629.28596	34.6819536	6.56183746
p21	6.45104349	0.46370076	1569.97092	1591.95415	630.053602	18.1557846	9.49470899
p-p21	6.48341516	0.5038796	1857.37495	4592.18497	107.793395	9.53785444	10.1113924
p27	6.37471234	0.9331044	718.065902	866.060381	2911.37616	44.2815305	10.1081081
p-p27	7.31495785	0.68156289	746.468132	551.025209	2665.77271	30.1878272	7.95336788
p38	6.46027583	0.31336035	727.846199	588.212965	5505.69919	54.531433	6.19736842
p-p38	6.5446979	0.09138093	983.501078	2071.57046	700.793202	24.828498	5.57377049
p-ERK	6.44468899	0.70707617	913.976836	890.949155	2365.7115	37.6924918	6.92015209
Cyclin B1	6.64415963	0.50225685	912.835678	826.054839	3050.90059	43.6578988	5.60128617
IFN-y	6.45587552	0.34102307	826.167115	544.492649	4009.04013	36.653937	6.69262295
biotin IFN-y	6.42640065	0.13482229	1244.56755	1613.80366	563.224489	18.281687	8.80906149
6A7 Bax	6.69588997	0.75509245	912.086591	1216.86118	2346.99294	45.0626643	5.07854985
Anti-BrdU	7.87462317	0.20935228	2624.61493	6649.98001	51.3294253	6.52958368	31.1350575
SR 1H4	6.43766881	0.66607249	763.409327	685.3969	4534.99939	52.4777171	6.48695652
Clk1	6.45874743	0.8520575	759.433941	618.395681	5340.39725	54.3463068	6
LnkB B2	6.76805579	0.14024813	759.700558	496.349923	578.518773	9.01597547	10.6933333
**Antibody**	**CIM330K long**	**CIM330K long**	**CIM330K long**	**CIM330K long**	**CIM330K long**	**CIM330K long**	**CIM330K long**
	Entropy	95^th^%ile var	mean	stdev	kurtosis	skewness	DR
AKT1(B-1)	6.59699095	0.28557628	4554.77322	1300.73519	622.307065	13.5654982	2.14203317
AKT1(7)	6.40821724	0.21273616	4104.46845	873.168854	838.54933	14.029646	1.84681881
Akt2(D17)	6.34880064	0.1995204	4411.52709	880.18965	1240.02572	19.8666298	1.69823386
p53 Ab1	6.43404063	0.24515575	4647.1139	1139.2667	1048.33146	21.9729809	1.73474044
p53 Ab8	6.67474666	0.3634331	4424.55606	1608.03013	286.62671	11.3306855	2.10854583
DM1A	6.58937783	0.36094758	4415.52644	1593.77357	563.688905	15.318917	2.61970256
JNK2	6.57656755	0.32165721	4352.02409	1399.85992	506.28591	12.0138395	2.74750357
hnRNP-A1	7.00998041	0.38475586	5347.05528	2057.31086	93.9357374	5.06685171	2.65914319
FDFT1	6.5936509	0.29834674	4274.13257	1275.17352	361.444301	7.32255294	2.7878202
p21	6.45104349	0.24655935	4927.17232	1214.84041	893.585872	21.018091	1.69355698
p-p21	6.48341516	0.44809185	4725.45688	2117.43872	648.486626	23.0610095	1.78139269
p27	6.37471234	0.211287	4191.46674	885.602419	1198.53625	18.8510712	1.77690289
p-p27	7.31495785	0.37818719	6757.56846	2555.62585	189.910928	9.03702438	2.4589372
p38	6.46027583	0.25272441	3713.48359	938.487955	1043.38512	16.7504264	2.06378601
p-p38	6.5446979	0.24457169	4408.19167	1078.1189	802.593762	16.2704786	1.94568151
p-ERK	6.44468899	0.20026886	4262.81051	853.708201	381.353761	7.32271088	1.84014502
Cyclin B1	6.64415963	0.30382729	4395.02676	1335.32909	336.369724	7.53027933	2.883585
IFN-y	6.45587552	0.20864676	4468.47338	932.332503	208.361855	5.46462466	1.81229385
biotin IFNy	6.42640065	0.21276461	4075.07667	867.03208	443.965119	8.29371879	1.86209262
6A7 Bax	6.69588997	0.28923639	4457.08591	1289.15144	389.869412	8.34131925	2.77285319
Anti-BrdU	7.87462317	1.04819122	9360.77698	9811.88428	17.6028326	3.91314999	6.89076795
SR 1H4	6.43766881	0.28279005	3832.10196	1083.68032	1228.81542	23.5580461	1.95700576
Clk1	6.45874743	0.20236299	4026.64226	814.843357	126.800971	2.54476741	1.92880376
LnkB B2	6.76805579	0.32786603	4695.37197	1539.45295	418.000148	12.2604133	2.22320025
**Antibody**	**HT124K**	**HT124K**	**HT124K**	**HT124K**	**HT124K**	**HT124K**	**HT124K**
	Entropy	95^th^%ile var	mean	stdev	kurtosis	skewness	DR
AKT1(B-1)	3.6388709	2.12000013	172.084868	364.819943	26306.4389	154.975997	2.53465347
AKT1(7)	4.33674097	1.01457968	236.252576	239.697063	57.2347386	5.49309364	6.84210526
Akt2(D17)	3.09156101	7.96233657	99.5903922	792.972222	5887.25715	74.611924	2.87755102
p53 Ab1	4.09079536	2.71704953	260.076266	706.640097	7458.35971	83.2355533	2.8707483
p53 Ab8	5.39118295	1.56358319	666.653349	1042.36797	428.188284	12.1835834	11.33
DM1A	3.72867736	2.82387858	142.715166	403.010301	18058.3269	127.906169	3.92753623
JNK2	3.05772087	4.98656856	94.1531951	469.501363	17414.857	129.406235	2.64150943
hnRNP-A1	3.74197272	1.10982143	169.803894	188.452	23264.3056	121.881413	3.09782609
FDFT1	2.87791212	0.45522638	78.6092266	35.7849934	6202.12906	48.1048494	2.55319149
p21	4.88056159	1.68084853	393.470917	661.365011	4580.52622	48.8499872	9.21804511
p-p21	3.56907199	0.45462944	152.14079	69.1676822	149.452867	8.20823774	2.64044944
p27	3.8794242	0.82617176	169.275086	139.850295	6939.03407	44.9492098	4.17647059
p-p27	3.18314358	2.5335869	97.6363798	247.370252	53415.3941	221.336524	2.7962963
p38	3.50561153	0.65966101	141.976199	93.6561625	20155.9907	96.2479979	2.71084337
p-p38	3.64849878	1.7776175	161.28086	286.695679	37134.4212	182.895648	2.7311828
p-ERK	4.61384818	1.2271232	291.994153	358.3128	10823.1069	68.0910883	6.7800885
Cyclin B1	3.26447123	2.69397794	104.030158	280.25495	36949.8652	182.207299	3
IFN-y	4.8490326	1.63623531	415.251753	679.449578	56.9535342	6.07266769	11.7723577
biotin IFNy	4.7279444	1.39308613	337.57957	470.277415	3010.19085	25.3004702	10.2293578
6A7 Bax	4.44449254	1.48090943	230.309572	341.067618	6035.3077	57.5689551	8.11392405
Anti-BrdU	3.63407385	3.90077997	129.479389	505.070606	14506.1405	117.222898	3.67741935
SR 1H4	3.2696181	1.79397273	131.299509	235.547738	51648.0352	205.542601	2.26829268
Clk1	3.11953784	7.31745995	126.44449	925.252493	4679.6103	67.7634574	2.26760563
LnkB B2	3.50192613	1.38543046	143.405965	198.678993	94781.8251	288.355356	2.67073171
**Antibody**	**CIM125K**	**CIM125K**	**CIM125K**	**CIM125K**	**CIM125K**	**CIM125K**	**CIM125K**
	Entropy	95^th^%ile var	mean	stdev	kurtosis	skewness	DR
AKT1(B-1)	2.7304762	9.56982994	45.2389304	432.92887	21488.5224	144.901688	21.0285445
AKT1(7)	3.96246833	2.19201832	126.868797	278.098727	24682.4368	114.187669	23.6168285
Akt2(D17)	2.60484067	8.25812471	43.3103286	357.662095	29163.4835	166.275502	20.6748661
p53 Ab1	2.80146844	2.8860048	44.1978863	127.555312	45700.074	189.930883	22.4974253
p53 Ab8	3.26418745	4.88039295	68.4303708	333.967099	34156.1048	178.224829	19.630186
DM1A	2.88766904	1.64499373	53.2250516	87.5548762	12896.8206	73.1321188	27.4205021
JNK2	2.98492852	1.89638275	54.1953277	102.775084	15832.7989	99.6436487	20.361336
hnRNP-A1	3.50630798	2.41101314	90.1888438	217.446487	62303.2699	207.955481	28.9977876
FDFT1	3.1782642	3.38281418	74.1013955	250.671251	43175.8734	180.552818	17.1889727
p21	4.21970354	3.3232106	268.050998	890.789917	1227.63171	22.8112489	19.184719
p-p21	3.39455331	1.19360386	78.1222364	93.2470027	7338.86079	45.3496167	30.2004608
p27	4.55717389	2.17699657	250.41672	545.15634	212.4138	10.4702714	14.9222973
p-p27	4.61752958	2.11107401	248.856763	525.355046	7398.48255	62.1267913	14.3288193
p38	3.4471677	3.98325087	92.5377642	368.60113	12266.5409	91.6929588	27.7616279
p-p38	2.80426187	7.66093095	69.5453262	532.781941	12536.7642	105.415318	24.4554335
p-ERK	3.29293995	5.27297172	71.8309949	378.762805	19803.3187	130.168903	21.3205298
Cyclin B1	3.15329576	2.60616442	59.6286291	155.402012	93376.3096	281.279467	18.8500387
IFN-y	4.75572062	2.14758346	362.463633	778.420904	424.884236	9.97913068	16.8441989
biotin IFNy	2.77340883	14.9757514	68.9829021	1033.07079	3481.86931	58.0367062	20.5053191
6A7 Bax	4.16781141	4.05548264	240.430834	975.063073	2365.29238	38.5653819	26.9137577
Anti-BrdU	3.61209451	5.95437037	88.4579233	526.711238	12990.5474	110.402725	23.5296572
SR 1H4	3.00625783	1.5817682	56.1568498	88.8271193	24601.5136	108.767101	22.9143357
Clk1	2.28663955	3.19223835	33.3028729	106.310708	36342.3158	165.64905	25.9708353
LnkB B2	3.53470995	4.48848749	182.78519	820.429041	381.626817	15.7874471	17.5305079

**Fig 3:**
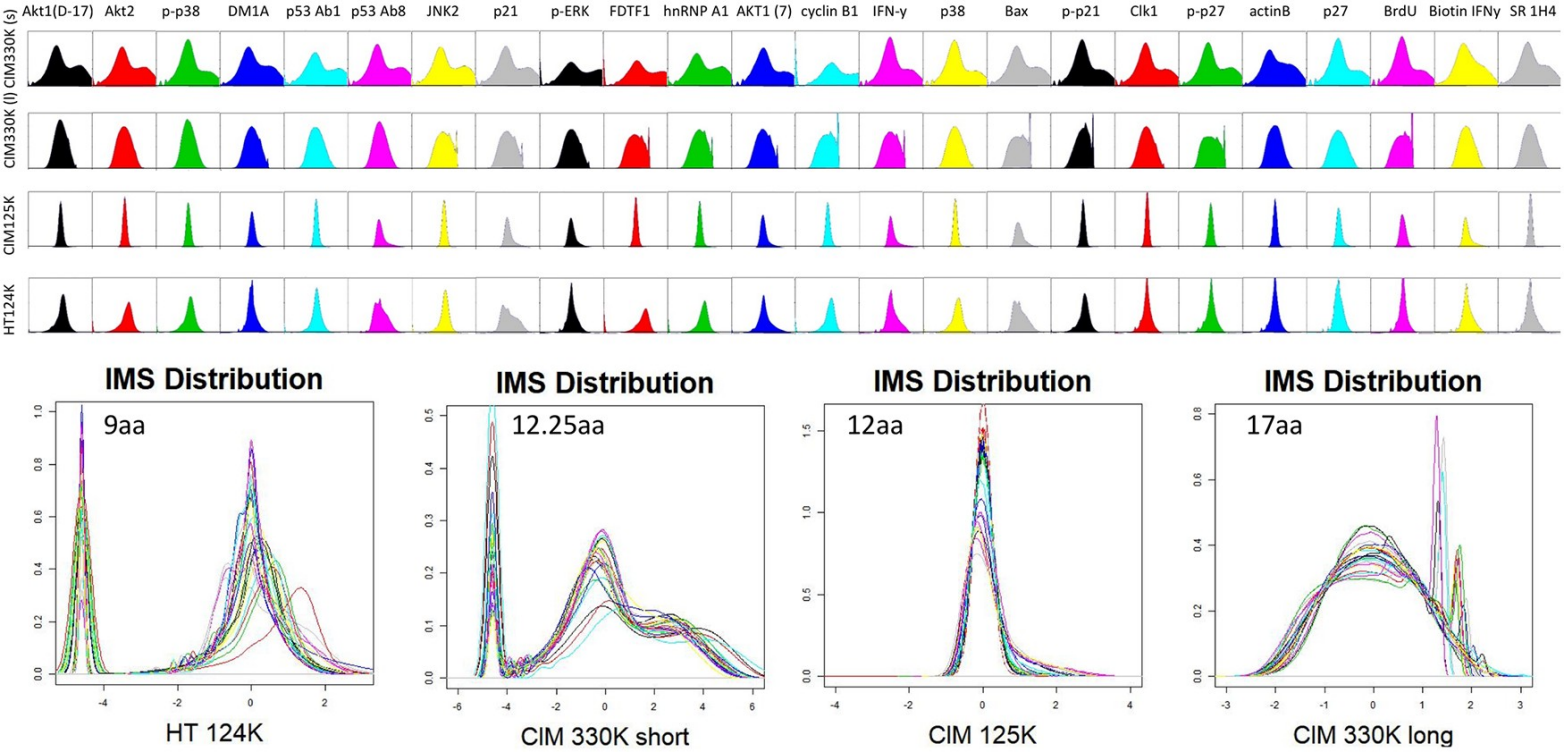
Data distribution for 24 monoclonals, for each peptide microarray library. Every peptide is shown as raw data for each of the 3 different peptide microarray libraries. Distribution kurtosis and 75^th^ percentile distributions are correlated to binding promiscuity.

Fig 3 shows the distributions plotted as density maps. It is easier to view these densities as general patterns rather than try to examine the finer details per distribution. As seen, there is substantial variation in the shape of the curves, and the breadth. The wider the distribution plot, the higher the dynamic range of the peptide binding scores. The plots also give an indication of normality, with some plots like hnRNP A1, p21, and BAX showing the most deviation from log_10_ normality on all libraries tested. Further, the CIM125K library and the HT124K library tended to produce distributions closer to log_10_ normality, but also provided less dynamic range.

In the lower half of Fig 3, the data distributions are plotted together, which highlights how few antibodies deviate from the general plot shape. For the libraries with variable peptide length, the distribution of lengths is plotted as a histogram within the density plots. The CIM330K short library has a wider distribution of lengths than the HT124K library. This effect is seen in the total number of peptides for a given length; the 330K library has a higher percentage of lengths at different discrete values relative to the mean length than the HT124K library, meaning that there is a wider range of peptide lengths.

**Fig 4:**
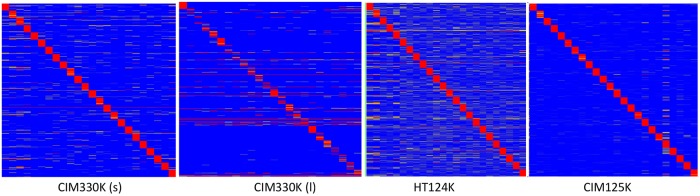
Hierarchical clustering of the top 50 peptides for each of the 24 monocloanls tested. The top 200 peptides for each monoclonal were selected by filtering via pattern-matching to a perfectly discriminatory pattern (i.e. high for each monoclonal, low for the other 23 monoclonals). This filter produced peptides that are unique to each monoclonal, if possible. The values for these 50*24 = 1200 peptides is shown for the three microarray libraries. The peptides were clustered using Pearson’s correlation coefficient to group peptides on the Y axis, the X axis lists each monoclonal, and was ordered manually Common reactivity is seen as colored bars off the diagonal axis.

Fig 4 is a heatmap showing the relationship of the binding intensity per peptide per monoclonal relative to those peptides binding to the other 23. This serves to highlight the specificity of the assay–for each monoclonal there are 200 peptides that bind only to that antibody and do not bind any other antibody.

To understand whether this method can provide information about unknown antibodies, we asked a collaborator for a collection of clinical monoclonals that were tested for their ability to remyelinate the central nervous system (CNS) in patients who were in remission from multiple sclerosis. These monoclonals were sourced from human Waldenstrom’s myeloma cells. Notably they are IgM rather than IgG. These were selected due to the properties of remyelinating activity *in vivo*. Their effects in laboratory mice infected with Theiler’s virus, a picornavirus that persists in the CNS and causes demyelination, demonstrated that no antibody stopped demyelination but they initiated remyelination of damaged neurons. This is the clinical effect that was being sought. If the immunosignature data from the commercial monoclonals is relevant, it should work for ANY monoclonal, not just well-characterized IgG molecules. We assayed the five human and five mouse antibodies, but were left blinded to their clinical data.

One of the antibodies (HIgM22) completed phase I clinical trials in multiple sclerosis patients without any serious complications. Whether this antibody that promotes remyelination in animals also promotes remyelination in multiple sclerosis patients is unknown. The dataset was analyzed blinded and reported to the collaborator who then interpreted the IMS data relative to each antibodies efficacy to promote CNS remyelination.

The concept that antibodies can promote remyelination comes from previous experiments in the Mayo/Rodriguez lab where adoptive transfer of antisera raised against purified mouse spinal cord homogenate was able to induce remyelination in animals with CNS demyelination induced by Theiler’s virus. As a result, spleens from those animals that produced this remyelinating antisera were fused to produce mouse monoclonal antibodies. These monoclonals were then screened for their ability to bind to CNS by immunofluorescence. Those that bound to myelin were then injected into mice infected with Theiler’s virus to determine which antibodies promote remyelination. It was shown that those antibodies that promote repair were polyreactive and had similar DNA sequences to germline making them natural antibodies. Once this was known, then human patients with monoclonal gammopathies were screened for their ability to bind the CNS myelin by immunofluorescence and then to promote remyelination in the Theiler’s virus model of demyelination. One of these antibodies that promoted consistent remyelination (rHIgM22) was sequenced and cloned to obtain a recombinant protein which is now being used in the multiple sclerosis clinical trial.

### Informatic analysis of 24 monoclonals

We first wished to test the general characteristics of the 24 monoclonals. Antibodies were assayed according to Materials and Methods. An image of 3 sample antibodies was taken from each of the three libraries. The 9 images are shown in Fig 1. Note that some antibodies have very restricted binding patterns, as exemplified by JNK2, where few peptides are bound by the monoclonal. This is the opposite of p21, where many peptides are bound by the monoclonal. Fig 2 shows a bar-chart of the 24 antibodies’ entropy calculation. Values range from 5 to 7 for all three libraries, but the range of values differs by the peptide library. The entropy scores should be compared non-parametrically using rank rather than absolute scores. The composition of the peptide library has a large effect on entropy, more than the differences across antibodies. Therefore each library should be analyzed relative to the antibodies tested, rather than a direct comparison of entropy across libraries. The highest entropy for all libraries was p21. The lowest for the two 125K libraries was actin B and phospho-p21. The lowest for the 330K library was anti-human p53 Ab8.

Fig 3 shows the density plots for each of the monoclonals. Density distributions reflect the binding variance and deviation from log-normal. As before, the CIM330K library shows some difference in the antibody profiles, suggesting that the older array may have properties unique to that library and synthesis method. The two newest array platforms show similar profiles.

Fig 4 is a display of the peptide intensities for 50 of the most unique peptides for each antibody. 1200 peptides are shown in total. As seen, there is little overlap in the peptides that each antibody bound well.

**Fig 5:**
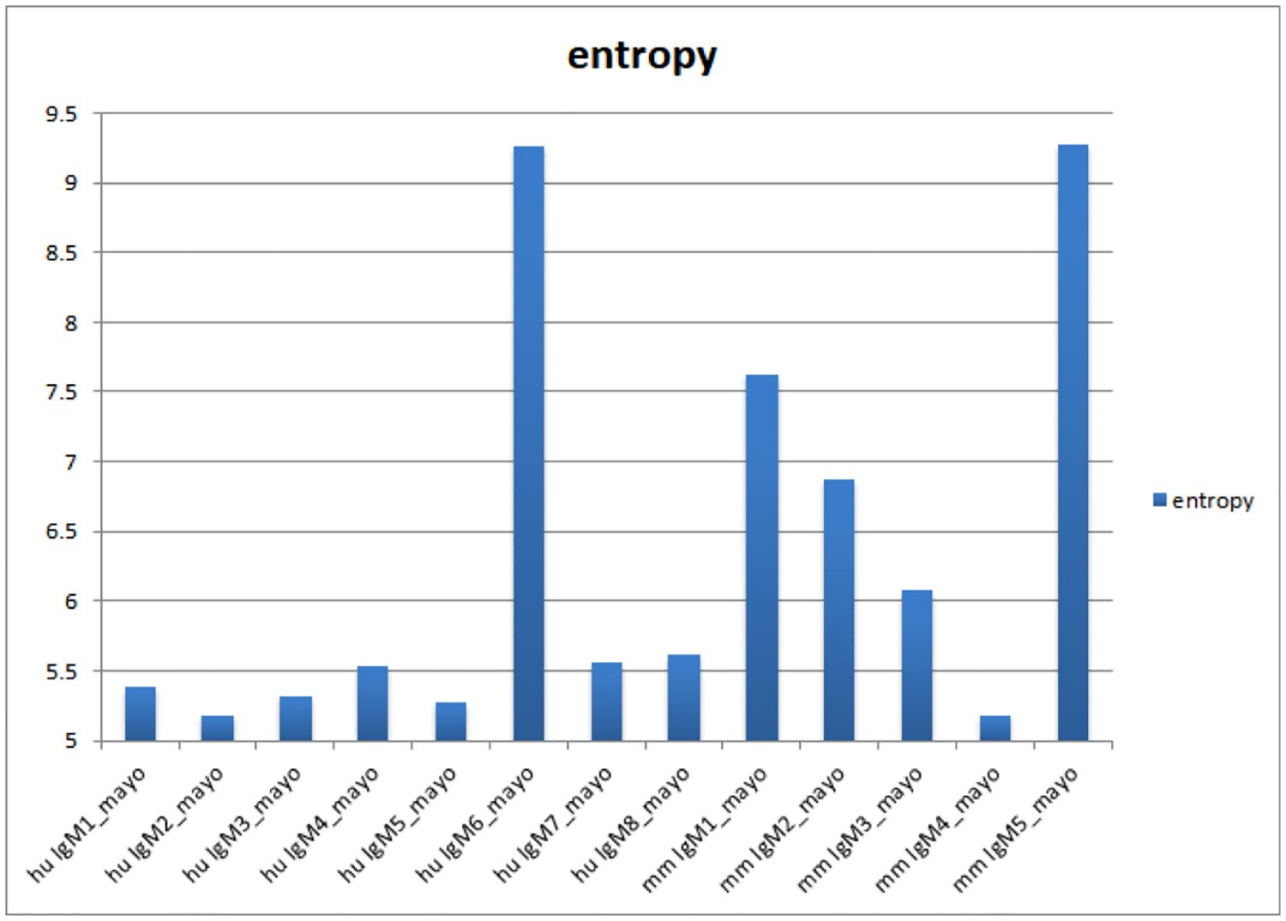
Entropy measures for 8 human therapeutic monoclonals and 5 mouse therapeutic monoclonals (IgM). Experimental IgM monoclonals used for therapeutic remyelination in human and mouse. IgM6 and IgM1 and IgM5 were shown by clinical trial significant efficacy in remyelinating human and mouse neurons, respectively. No other monoclonal showed efficacy.

Fig 5 is an analysis of the Shannon’s entropy score for the Mayo monoclonal antibodies. In this figure the highest entropy scores resulted from Human 6 and Mouse 5.

**Fig 6:**
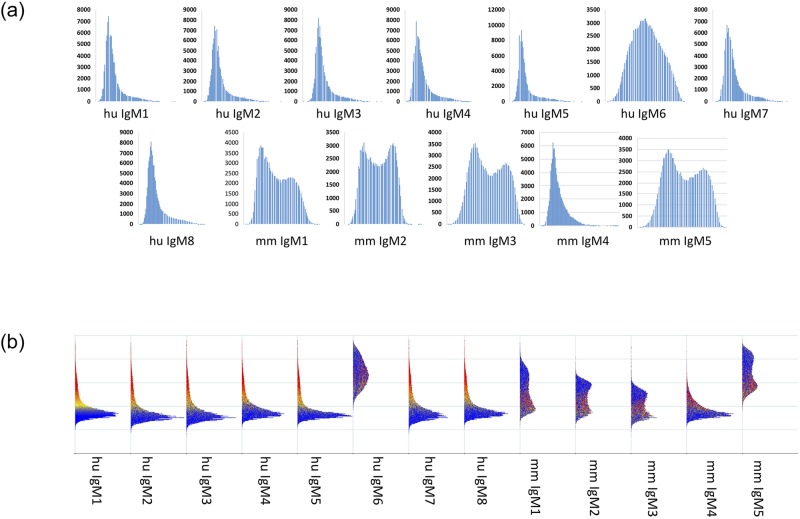
Data distribution for monoclonals shown in Fig 4. Data for all 125,000 peptides from CIM125K are shown as a density plot, either one by one (top plots) or side-by-side (bottom plot). For the distributions shown along the bottom, blue color indicates low intensity binding while yellow and red indicate higher binding at least above the median signal for that array. Antibodies are shown in the same order as Fig 4. Wide/broad distributions match promiscuous binding of specific antibodies, narrow distributions suggest more specific binding. No other serological test performed on any of these antibodies led investigators to predictions of efficacy, but therapeutic efficacy of these IgM antibodies correlated perfectly with the broad distributions and relatively high entropy scores.

In Fig 6 we see that these two monoclonals (IgMh6 and IgMm5) had the broadest distribution plots regardless of the method used to generate the smoothed histograms (Fig 6 top and Fig 6 bottom).

**Fig 7:**
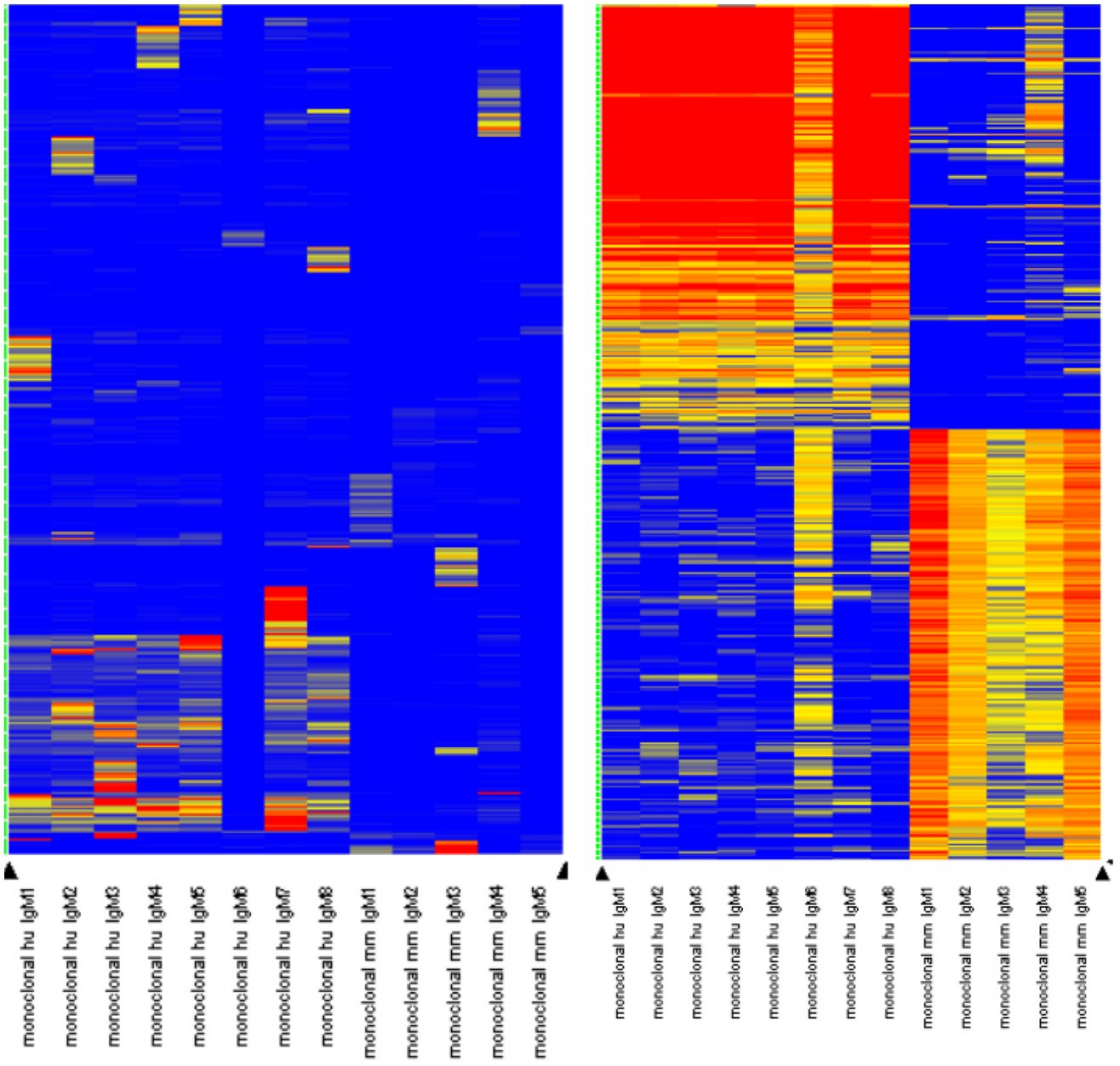
Heatmap for the 13 different clinical monoclonals. Each of the clinical monoclonals was tested exactly like the 24 commercial antibodies, to find 50 peptides that were unique for each antibody (see Fig 4). Left: For each of the antibodies, some unique peptides were identified but for the human antibodies, many peptides overlapped suggesting a common target. The mouse antibodies had less overlap with either the human or other mouse antibodies. Right: We applied a general filter for high binding peptides. Here there are 200 peptides identified for the human antibodies (left) and 200 for the mouse antibodies (right). These high-binding peptides overlap with each other, but not between mouse and human reinforcing the possibility that these two sets of antibodies are against different protein targets. These 200 peptides were used to BLAST all human and all mouse proteins, respectively (see Table 4).

In Fig 7 we show the results from the same method used previously to obtain peptides that bind uniquely to each monoclonal. The heatmap on the left shows that only 1/3 of the peptides selected to be uniquely bound by each monoclonal were in fact unique–the rest were generally common to all of the tested antibodies. This was most apparent in the mouse monoclonals (right side of heatmap). The heatmap on the far right shows the peptides that were selected for the human and the mouse antibodies. The common high binders were different for mouse and for human, suggesting that there were different epitopes being bound by the antibodies. These 200 peptides for mouse and separately 200 peptides for human were BLASTed against their respective proteomes using BLASTP with a low stringency E<0.01 cutoff. Each of the 200 peptides was used to obtain a list of matching proteins. For the human antibodies, there were 118 different protein targets found, for mouse there were 172 different proteins found. Table 5 lists the top four proteins that were found more often than any other protein. These four common proteins which were found both in mouse and human were identified by 400 different peptides. *We do not know the identity of the actual biological targets for these antibodies* but the common targets for both mouse and human appear to be cytoskeletal in nature. It is possible the size of these proteins plays a role in finding them using this probabilistic approach, but there are other large proteins in the human and mouse proteomes and both found similar proteins at approximately the same rate.

**Table 5:** List of most common hits from mouse and human peptides (from Fig 7). The Mayo monoclonals from Table 3 were tested on the 330K immunosignature array. 200 peptides that were common for the human and 200 peptides common for the mouse antibodies were used to compare the GeneBank human (hs) or mouse (mm) protein database using BLASTP and a cutoff of 0.01. The protein hits for each peptide were compiled and sorted. The table below contains proteins from both mouse and human that were hit at least 2-fold more often than the next most common protein. The first column lists the protein common name, the second column lists the number of times the 200 peptides aligned with each protein for human (column 2) and mouse (column 3). The next highest number of hits for human was 61 and for mouse was 47.

BLASTP hits	Human	Mouse
Kelch-like protein	123	126
Dynein heavy chain	535	768
Myosin family	350	322
Titin	202	88

**Fig 8:**
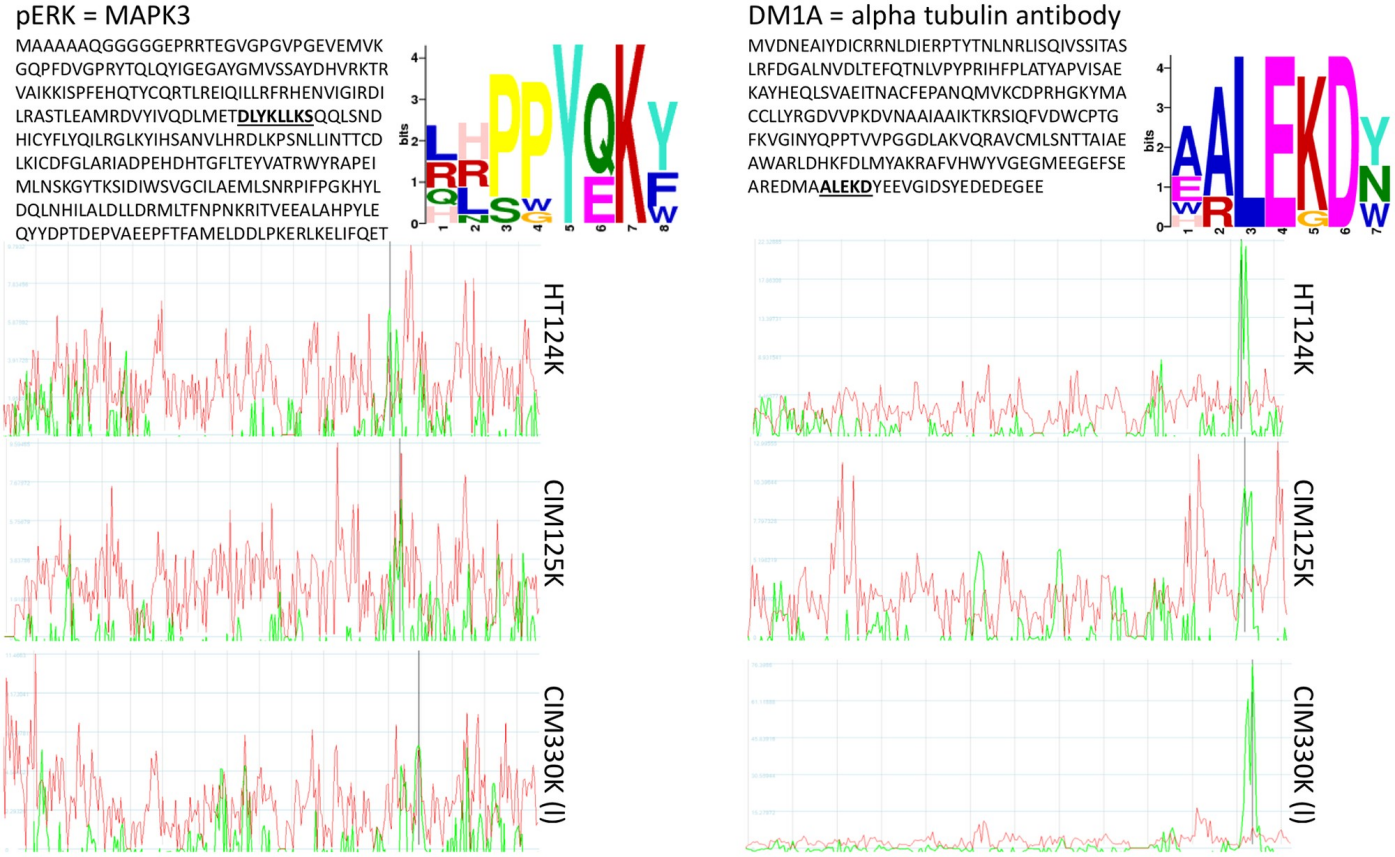
Alignment of peptide data from JNK2 (top) and DM1A (bottom). JNK2 and DM1A were processed on 3 microarray platforms. 125K, 124K and 330K array data were used to find epitopes using CLUSTALW and GLAM2. The large text figures represent the GLAM2 output. These motifs are similar to the actual linear epitope shown underlined in the protein sequence. Right: Guitope [13] was used to identify a region of either JNK2 (top three graphs) or tubulin (bottom three graphs). The red lines indicate the noise threshold, generated by testing all random peptides from each of the peptide libraries. The green line is the signal from the 200 selected peptides unique to that antibody. The black vertical line indicates the position within the protein where the epitope is likely to reside. For both proteins, the Guitope analysis predicted the exact location of the start of the epitope sequence. 72 residues from the C-terminus of JNK2 and 23 residues from the C-terminus of tubulin.

Fig 8 is an example of how a library of random-sequence peptides can identify a linear sequence of protein that defines the eliciting epitope, similar to the way standard epitope mapping experiments work. This relates to the previous analysis of the Mayo monoclonals suggesting that there may be some capacity of the random peptides to find actual epitopes from antibodies–we previously explored this capacity [12, 14, 16] with similar results.

## Discussion

We introduced a method for investigating binding properties of monoclonal antibodies. We examined the binding of 24 different commercially sourced monoclonal IgG antibodies to four different random-peptide immunosignature microarrays. Some monoclonals had published and defined targets, most only reported the protein as the immunogen. DM1A is a human anti-Tubulin IgG that was created by immunizing with the peptide AALEKDY while others like Akt1 antibody were raised to a segment of protein representing amino acids 345–480 in human Akt1. Some targets were not detailed by the manufacturer, a common practice for many commercial antibodies. We analyzed all but could not confirm whether our information about the target is correct. Some targets such as p-p21 were phosphorylated, and the non-phosphorylated pair, i.e. p-21, was also tested.

We first used a general exploratory analysis. We asked whether information theory could provide some insight into the behavior of the monoclonals. Here we used Shannon’s entropy [17] to explore how many independent binding events could be examined together to form a picture of antibody behavior. When examined along with the histograms of the data, a picture emerges of how a given antibody responds to random sequences. Some antibodies prefer to bind to many different peptides strongly, some to many peptides weakly, but most bound to several hundred to several thousand peptides strongly, regardless of the peptide library that was used. The 330K long array highlighted that some antibodies prefer to bind to longed peptides as evidenced by the increased number of high-binding events on the 330K long vs. the HT 124K library. A sequence analysis of the 200 highest binding peptides per antibody revealed that some monoclonals bound to similar motifs but those motifs could appear closer to the C-terminus (p53 Ab8) or to the N-terminus (p53 Ab1). These sorts of observations are difficult to obtain using classic antibody binding measurements like ELISA. Phage display can provide information like this, but unlike phage display, the immunosignatures can display non-binding information. P53Ab8 was raised to SDLWKLL and binds to peptides that differ substantially from this sequence as long as there is a tryptophan near the middle of the peptide. Almost no peptide with no tryptophan bound to p53 Ab8.

We then examined 13 IgM antibodies from Mayo. The molecular target for the remyelination antibodies is unknown. Neither the human nor mouse antibody panel has yielded a confirmed *in vivo* target. In Hecker et al. [18] a tiled peptide microarray made by JPT Peptides (Berlin, Germany) detected several candidate proteins with high antibody reactivity in relapsing remitting Multiple Sclerosis (RRMS); ACTB, ACTG (human actin B and actin gamma) were identified by several high-binding peptides in a majority of MS case samples. S100A1 and CRYAB are a heat shock protein and a calcium binding protein, respectively and were also identified by commonly binding peptides present in these proteins. Given the wide range of possible targets for these therapeutic remyelination antibodies, we followed an unbiased search using peptides that bound the monoclonals at the highest intensity.

Mayo provided the antibodies blinded. Analysis showed that IgMhu6 and IgMMm5 had the highest entropy values and the broadest and most non-normal density distributions. The patterns in Fig 7 indicate that there were few peptides that were completely unique to each antibody. The test to pick unique peptides is quite stringent as seen for the commercial antibodies in Fig 4. The human IgM antibodies showed a great deal of overlap, even as the selection process actively discouraged any overlap. This may suggest a common target. IgMhu6 showed little overlap with other antibodies. This may be due to many peptides binding simultaneously, decreasing specificity for a given set of peptides. The same pattern appears in mmIgM6. It is worth noting that the mouse antibodies had more specificity across the different clones than the humans. There was little commonality between the mouse antibodies in general vs. the human antibodies, as shown in Fig 8. Mayo demonstrated that Human 6 and Mouse 5 were most efficacious in promoting remyelination in animal models of demyelination, including both the Theiler’s virus model and direct lysolecithin injection into the cord. Mayo tested lipid panels, pull-downs, western blots, and other discovery methods, but the target remained elusive. Without a candidate protein, it is difficult to align motifs to obtain a confident target but using an *ab initio* approach we simply BLASTed the peptides against the human and mouse proteome, respectively. We identified Kelch-like (123 times in human and 126 times in mouse), dynein heavy chain (535hu and 768mm), myosin family protein (350hu and 322mm), and titin (202hu and 88mm). It may be that these overlapping proteins contain the target sequence from another protein, or the repeating units enhanced off-target alignments, but equally likely the Mayo antibodies are actually binding or stabilizing certain cytoskeletal components allowing remyelination. Dynein had hits along the length of the protein, but dynein is a large protein (nearly 5000 amino acids); peptides are likely to match it by random chance. However, a western blot study of these proteins might prove informative.

## Conclusions

The data provided here can be applied to any antibody. Epitope binning is a first and important characterization of therapeutic antibodies, but it may be that immunosignature analysis might provide insights not available with standard techniques. For example, information about which peptide sequences bind can reveal motifs like the actual epitope. However, information about which peptides ablate binding can be as important. Single residue changes that reverse a strong binder to a weak binder reveals a great deal about the paratope and the epitope determinant. Information about promiscuity or polyreactivity can be obtained in a single experiment on an immunosignature microarray. These facets of antibody character, as demonstrated here, could profoundly affect clinical efficacy such as promoting remyelination. A single value, entropy, could be used as a proxy for polyreactivity.

There are several immediate practical benefits that arise from this study. First, epitope binning is time consuming. It may be that a rapid screen with a random peptide microarray can narrow thousands of candidate monoclonals to a few that can be investigated more thoroughly. Polyreactivity was a strong indicator of clinical efficacy in this case, but there can be many different outputs. The breadth of data provided by immunosignatures lends itself to machine learning. By training an algorithm on data from successful or clinically useful monoclonals, that pattern, no matter how complex or convoluted, can be captured by sophisticated machine learning analyses in a high-throughput manner. This increase in speed is the key to increasing our ability to screen thousands or millions of antibodies, one of which could be the next major blockbuster.

## Supporting information

S1 File (PPTX)
