# Supplementary material for: Antibody characterization using immunosignatures
Source: PLoS One. 2020 Mar 20;15(3):e0229080. doi: 10.1371/journal.pone.0229080 (PMC7083272; doi:10.1371/journal.pone.0229080)
Supplement: S1 File — (PPTX) [file pone.0229080.s001.pptx]

## Slide 1
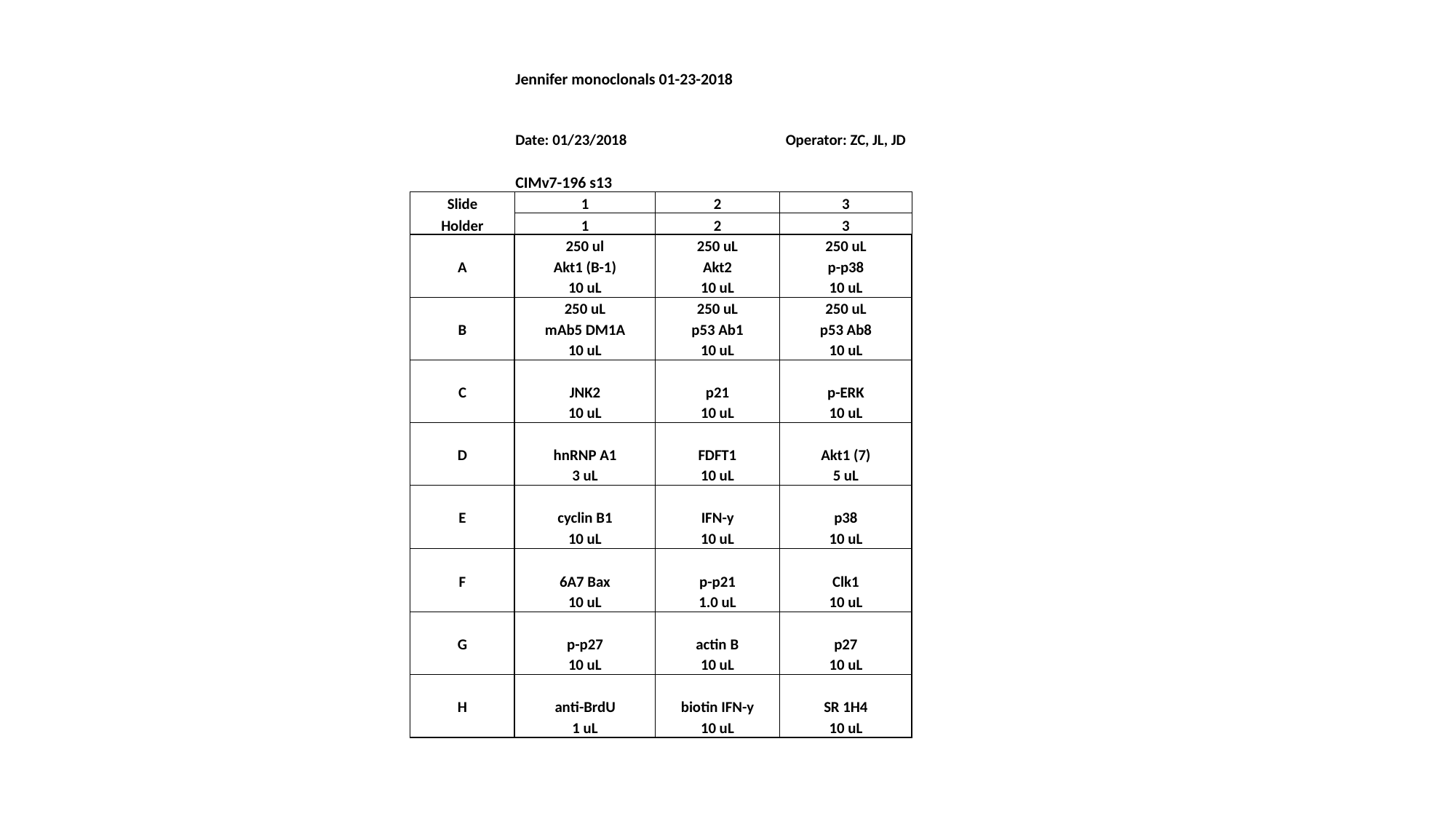

| | Jennifer monoclonals 01-23-2018 | | |
| --- | --- | --- | --- |
| | | | |
| | Date: 01/23/2018 | | Operator: ZC, JL, JD |
| | CIMv7-196 s13 | | |
| Slide | 1 | 2 | 3 |
| Holder | 1 | 2 | 3 |
| | 250 ul | 250 uL | 250 uL |
| A | Akt1 (B-1) | Akt2 | p-p38 |
| | 10 uL | 10 uL | 10 uL |
| | 250 uL | 250 uL | 250 uL |
| B | mAb5 DM1A | p53 Ab1 | p53 Ab8 |
| | 10 uL | 10 uL | 10 uL |
| | | | |
| C | JNK2 | p21 | p-ERK |
| | 10 uL | 10 uL | 10 uL |
| | | | |
| D | hnRNP A1 | FDFT1 | Akt1 (7) |
| | 3 uL | 10 uL | 5 uL |
| | | | |
| E | cyclin B1 | IFN-y | p38 |
| | 10 uL | 10 uL | 10 uL |
| | | | |
| F | 6A7 Bax | p-p21 | Clk1 |
| | 10 uL | 1.0 uL | 10 uL |
| | | | |
| G | p-p27 | actin B | p27 |
| | 10 uL | 10 uL | 10 uL |
| | | | |
| H | anti-BrdU | biotin IFN-y | SR 1H4 |
| | 1 uL | 10 uL | 10 uL |

## Slide 2
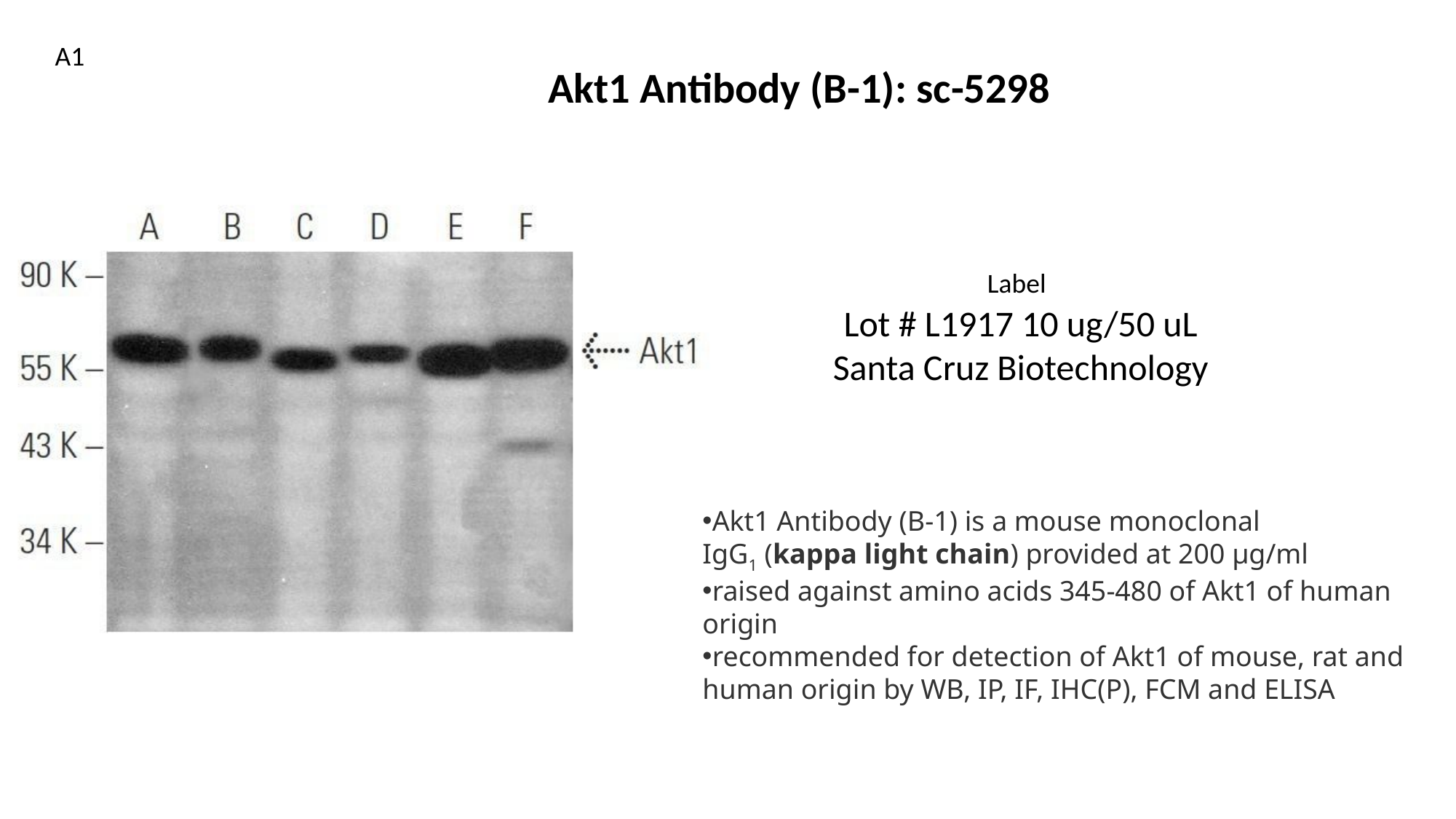

A1
Akt1 Antibody (B-1): sc-5298
Label
Lot # L1917 10 ug/50 uL
Santa Cruz Biotechnology
Akt1 Antibody (B-1) is a mouse monoclonal IgG1 (kappa light chain) provided at 200 µg/ml
raised against amino acids 345-480 of Akt1 of human origin
recommended for detection of Akt1 of mouse, rat and human origin by WB, IP, IF, IHC(P), FCM and ELISA

## Slide 3
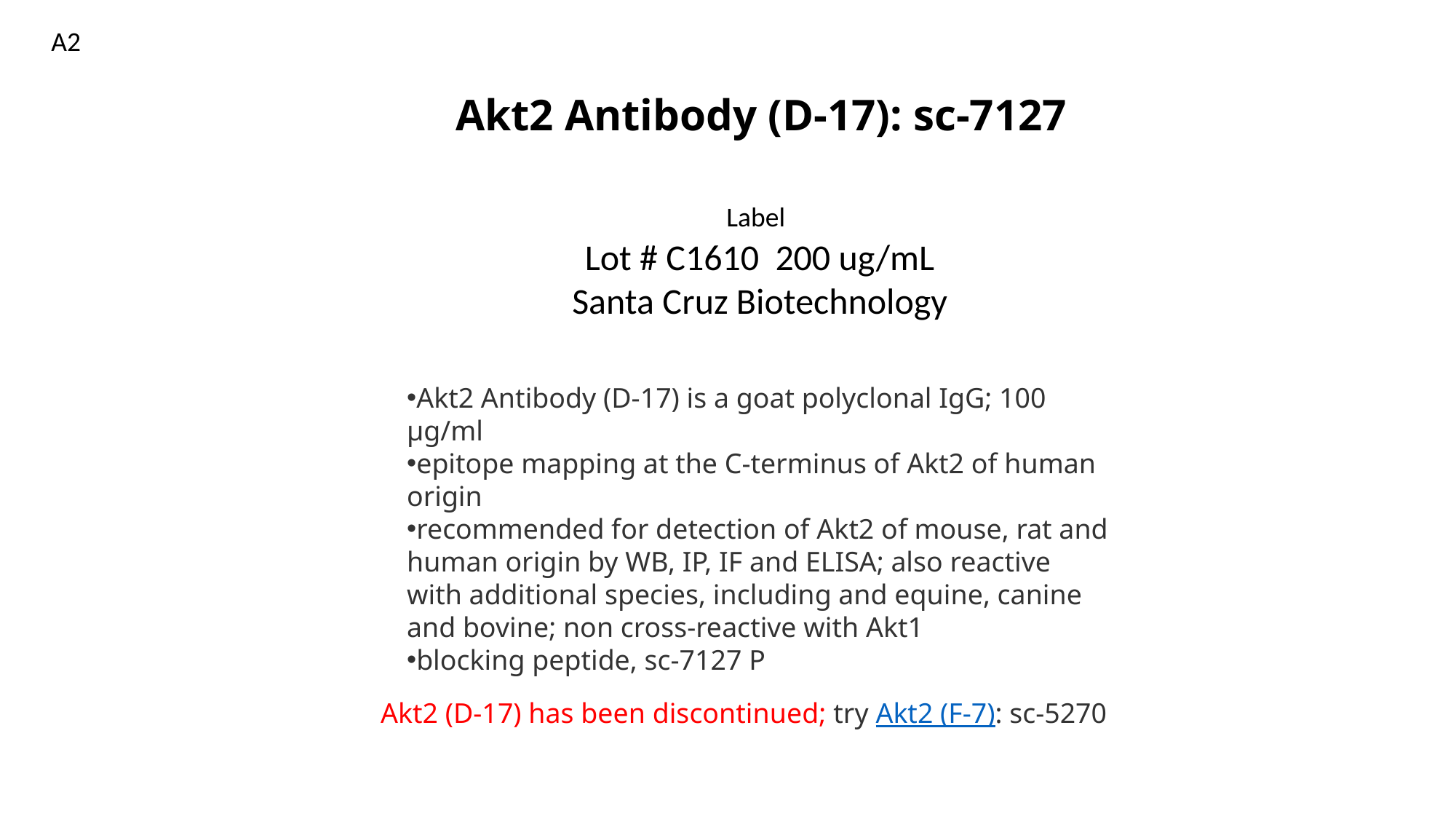

A2
Akt2 Antibody (D-17): sc-7127
Label
Lot # C1610 200 ug/mL
Santa Cruz Biotechnology
Akt2 Antibody (D-17) is a goat polyclonal IgG; 100 µg/ml
epitope mapping at the C-terminus of Akt2 of human origin
recommended for detection of Akt2 of mouse, rat and human origin by WB, IP, IF and ELISA; also reactive with additional species, including and equine, canine and bovine; non cross-reactive with Akt1
blocking peptide, sc-7127 P
Akt2 (D-17) has been discontinued; try Akt2 (F-7): sc-5270

## Slide 4
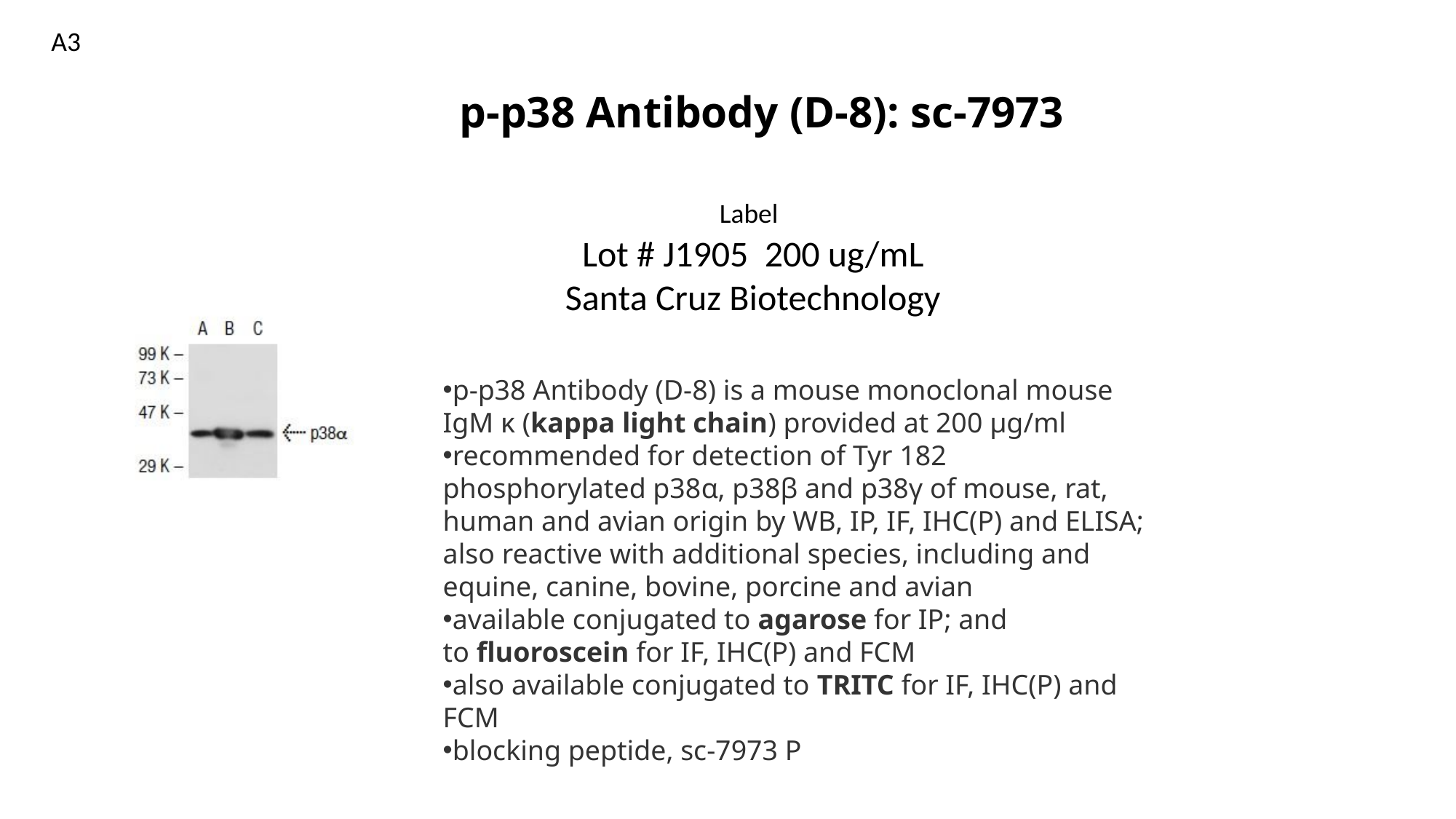

A3
p-p38 Antibody (D-8): sc-7973
Label
Lot # J1905 200 ug/mL
Santa Cruz Biotechnology
p-p38 Antibody (D-8) is a mouse monoclonal mouse IgM κ (kappa light chain) provided at 200 µg/ml
recommended for detection of Tyr 182 phosphorylated p38α, p38β and p38γ of mouse, rat, human and avian origin by WB, IP, IF, IHC(P) and ELISA; also reactive with additional species, including and equine, canine, bovine, porcine and avian
available conjugated to agarose for IP; and to fluoroscein for IF, IHC(P) and FCM
also available conjugated to TRITC for IF, IHC(P) and FCM
blocking peptide, sc-7973 P

## Slide 5
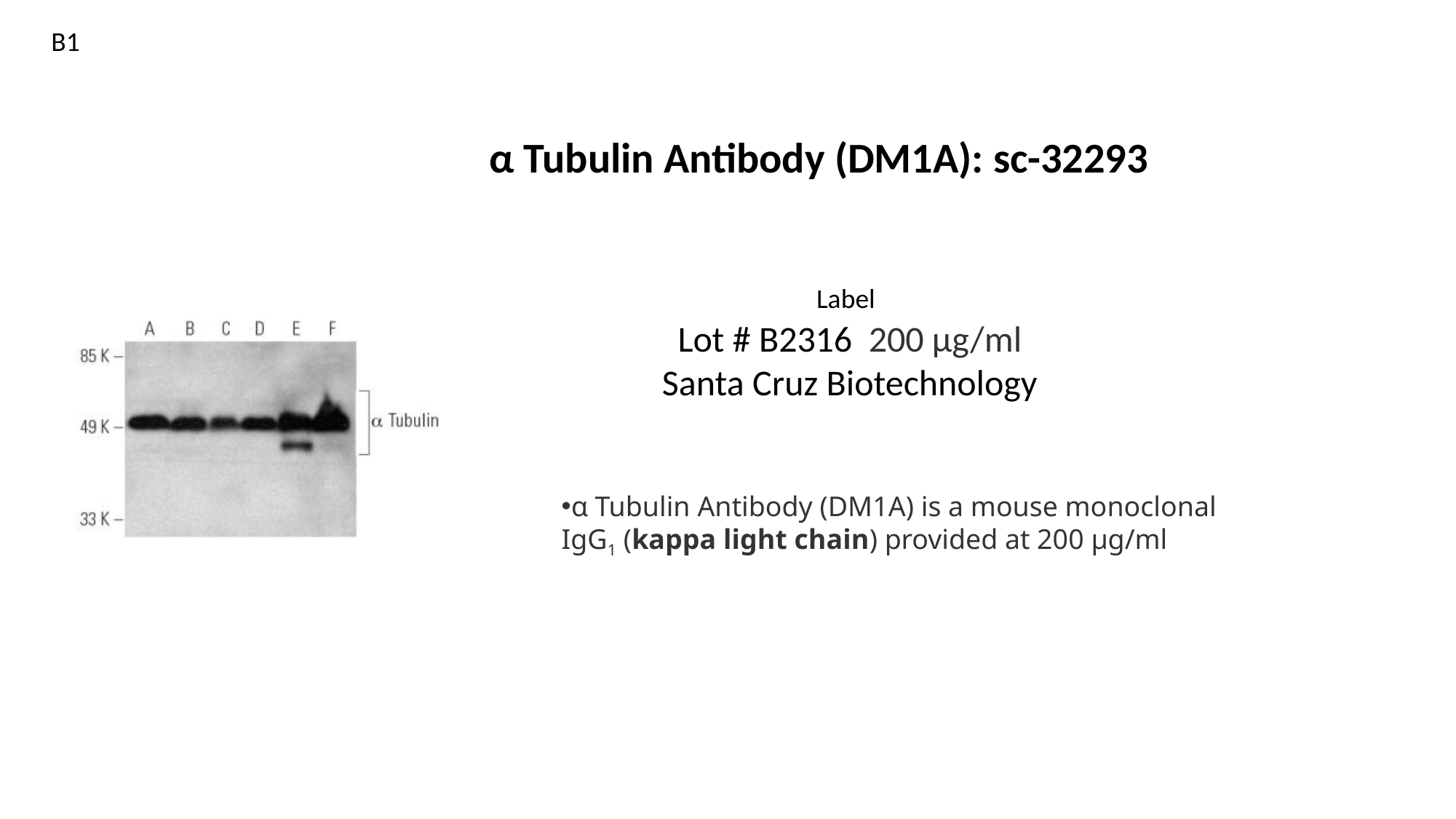

B1
α Tubulin Antibody (DM1A): sc-32293
Label
Lot # B2316 200 µg/ml
Santa Cruz Biotechnology
α Tubulin Antibody (DM1A) is a mouse monoclonal IgG1 (kappa light chain) provided at 200 µg/ml

## Slide 6
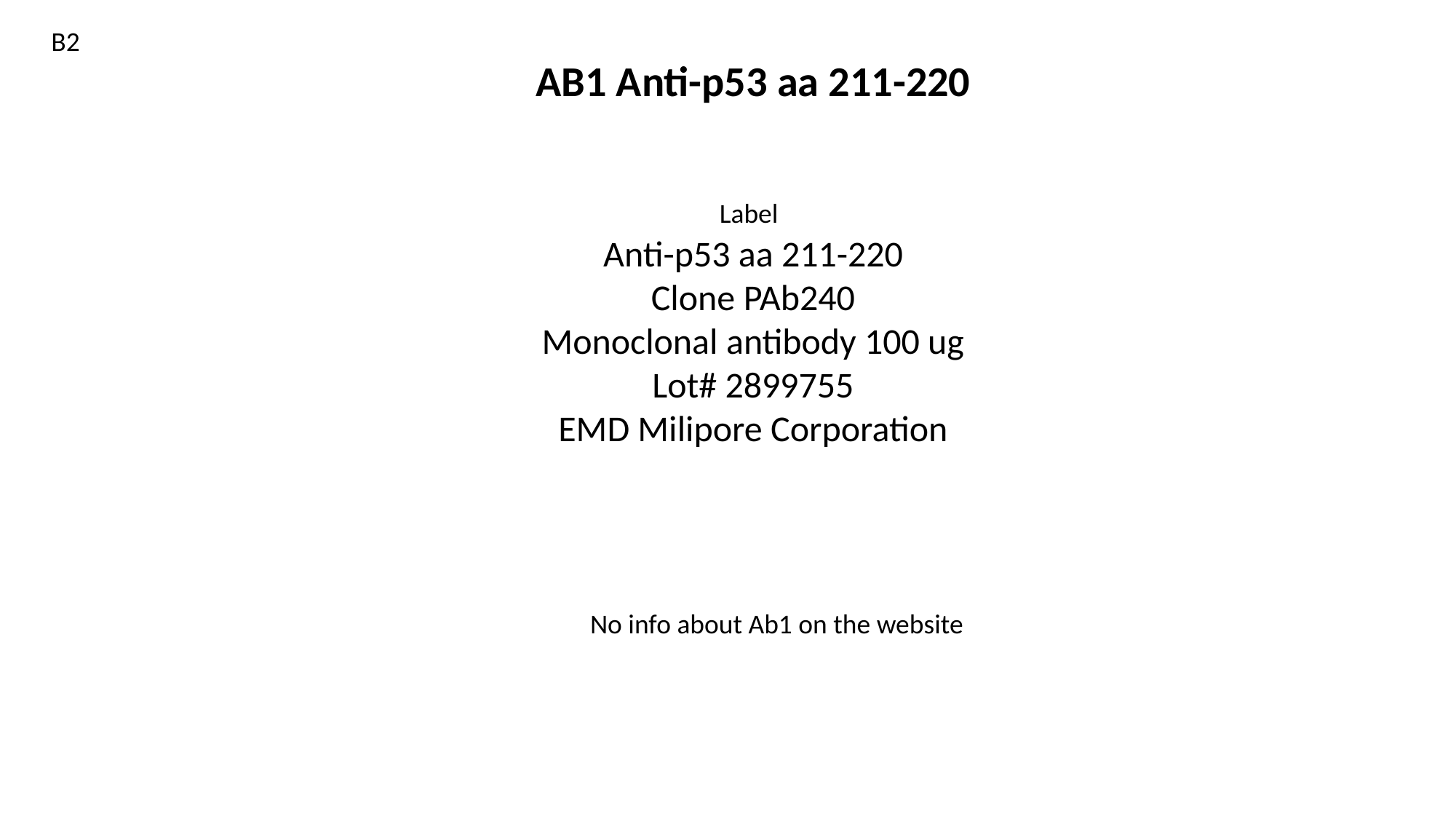

B2
AB1 Anti-p53 aa 211-220
Label
Anti-p53 aa 211-220
Clone PAb240
Monoclonal antibody 100 ug
Lot# 2899755
EMD Milipore Corporation
No info about Ab1 on the website

## Slide 7
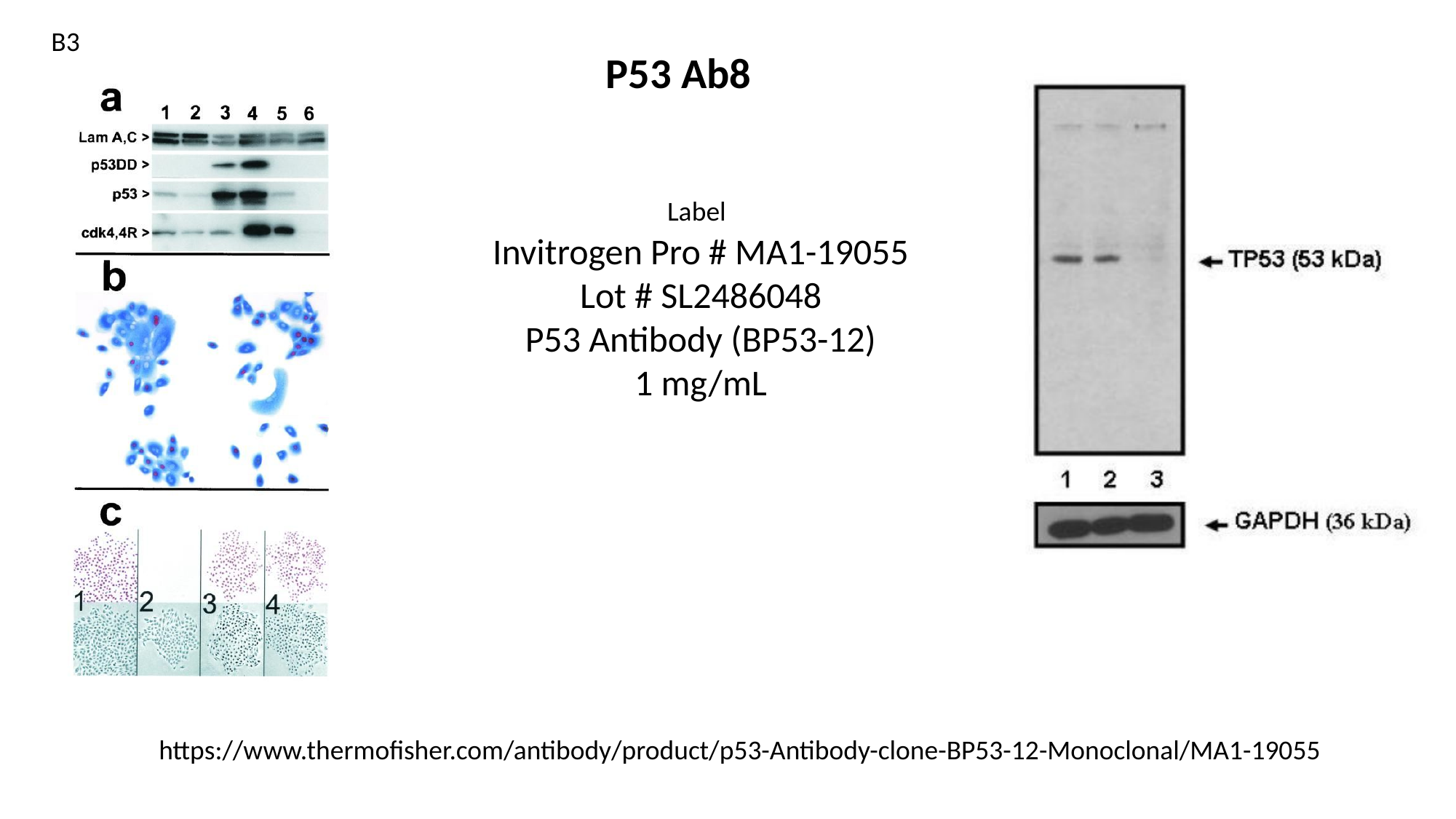

B3
P53 Ab8
Label
Invitrogen Pro # MA1-19055
Lot # SL2486048
P53 Antibody (BP53-12)
1 mg/mL
https://www.thermofisher.com/antibody/product/p53-Antibody-clone-BP53-12-Monoclonal/MA1-19055

## Slide 8
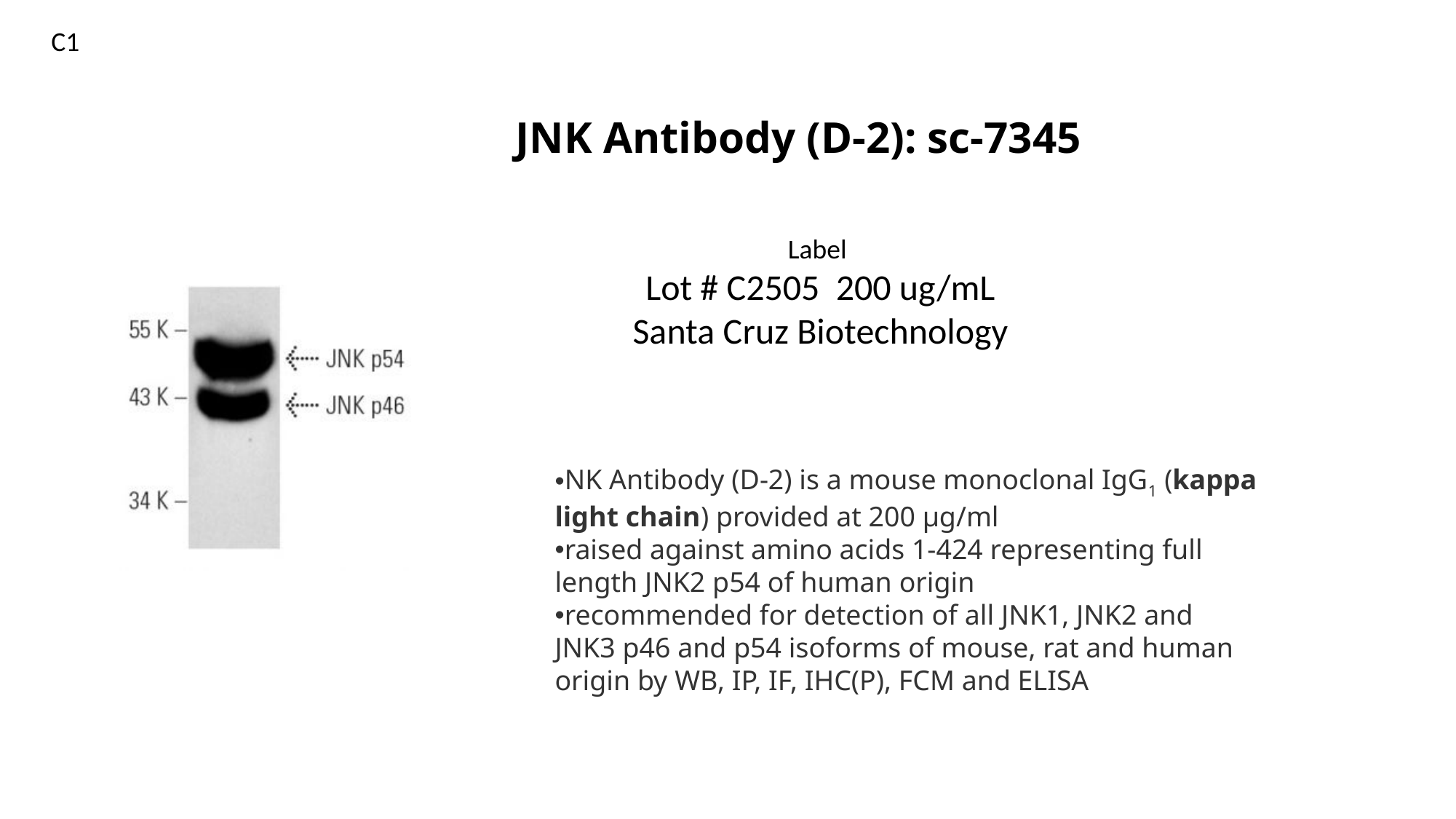

C1
JNK Antibody (D-2): sc-7345
Label
Lot # C2505 200 ug/mL
Santa Cruz Biotechnology
NK Antibody (D-2) is a mouse monoclonal IgG1 (kappa light chain) provided at 200 µg/ml
raised against amino acids 1-424 representing full length JNK2 p54 of human origin
recommended for detection of all JNK1, JNK2 and JNK3 p46 and p54 isoforms of mouse, rat and human origin by WB, IP, IF, IHC(P), FCM and ELISA

## Slide 9
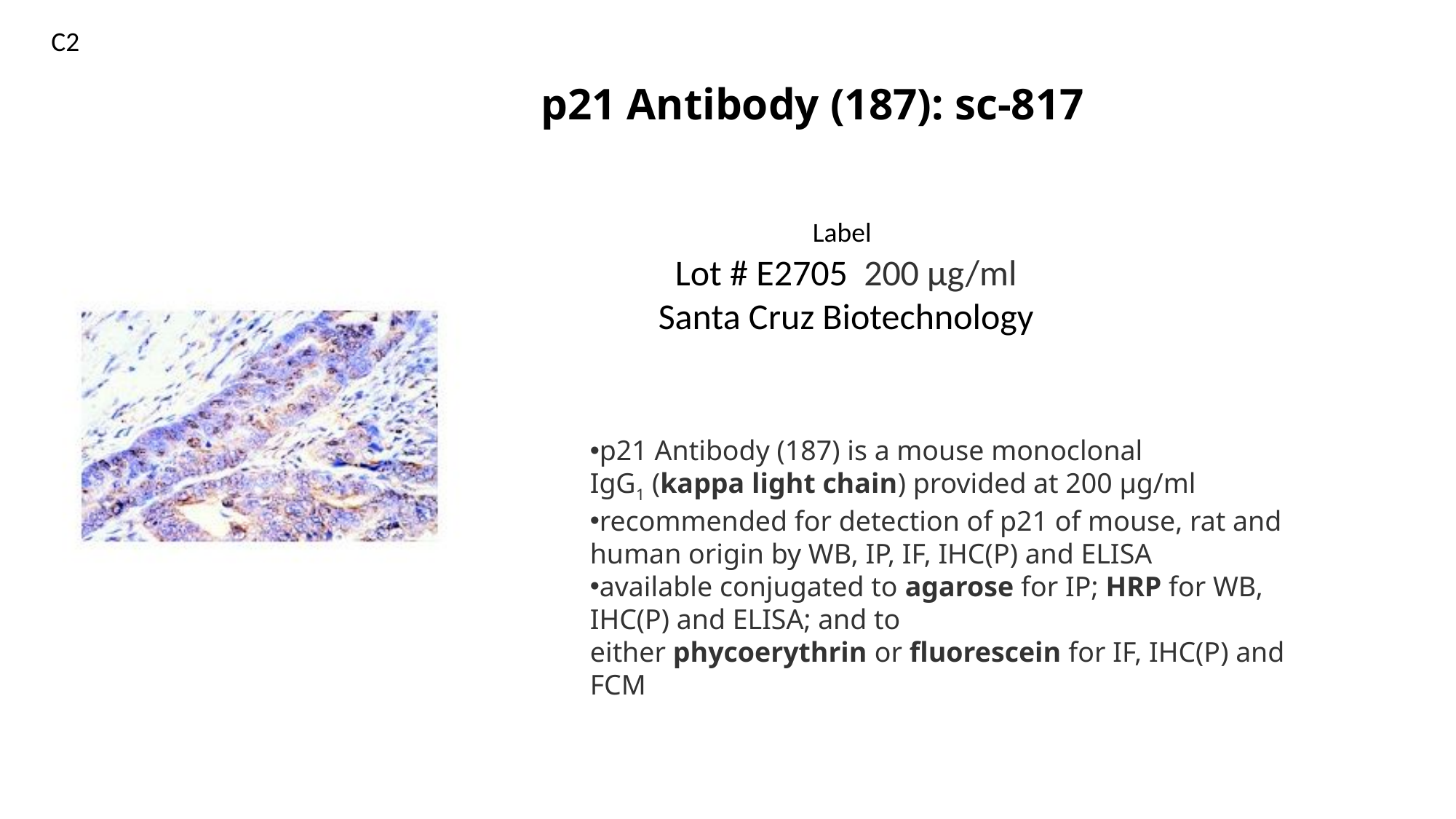

C2
p21 Antibody (187): sc-817
Label
Lot # E2705 200 µg/ml
Santa Cruz Biotechnology
p21 Antibody (187) is a mouse monoclonal IgG1 (kappa light chain) provided at 200 µg/ml
recommended for detection of p21 of mouse, rat and human origin by WB, IP, IF, IHC(P) and ELISA
available conjugated to agarose for IP; HRP for WB, IHC(P) and ELISA; and to either phycoerythrin or fluorescein for IF, IHC(P) and FCM

## Slide 10
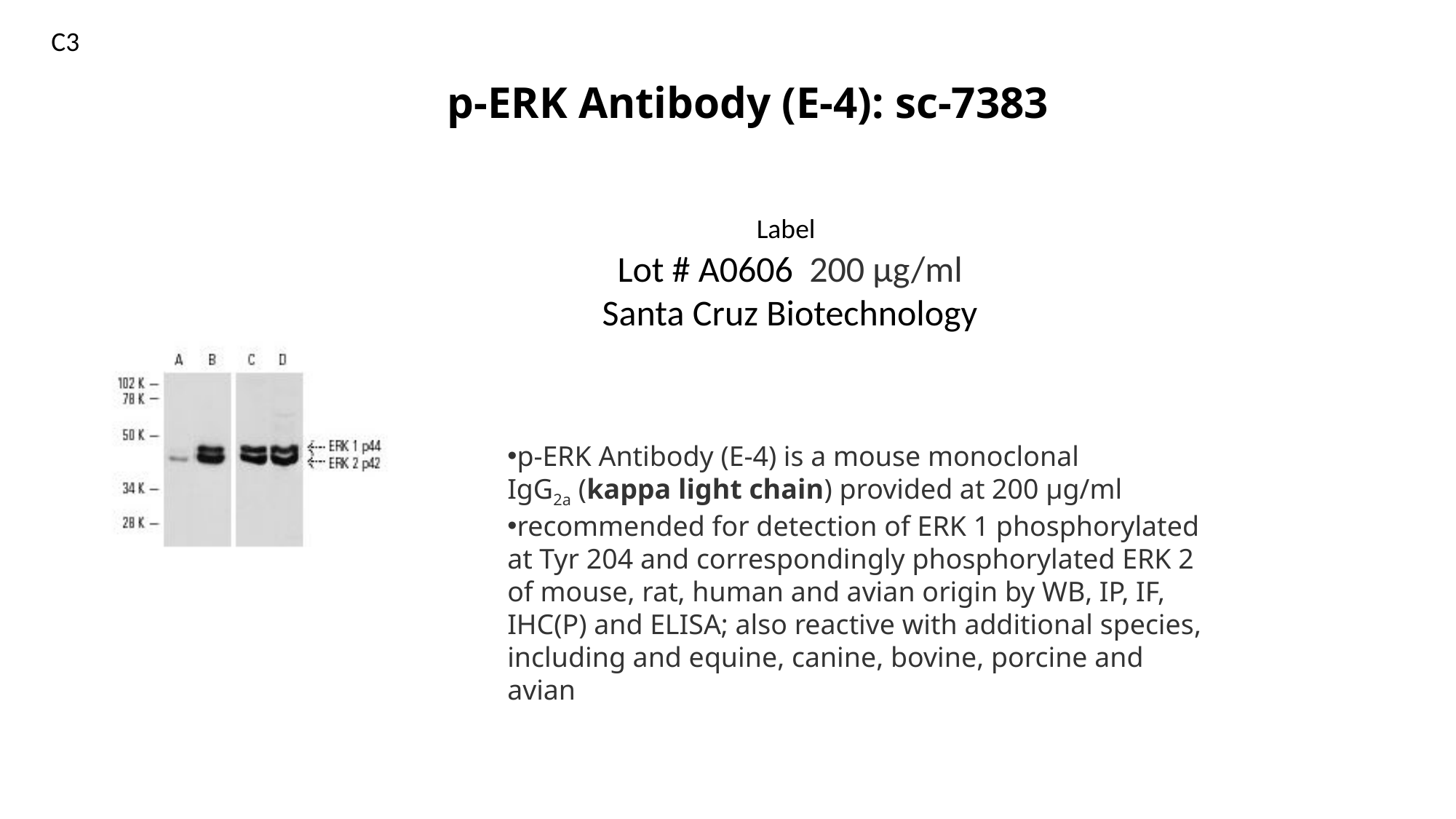

C3
p-ERK Antibody (E-4): sc-7383
Label
Lot # A0606 200 µg/ml
Santa Cruz Biotechnology
p-ERK Antibody (E-4) is a mouse monoclonal IgG2a (kappa light chain) provided at 200 µg/ml
recommended for detection of ERK 1 phosphorylated at Tyr 204 and correspondingly phosphorylated ERK 2 of mouse, rat, human and avian origin by WB, IP, IF, IHC(P) and ELISA; also reactive with additional species, including and equine, canine, bovine, porcine and avian

## Slide 11
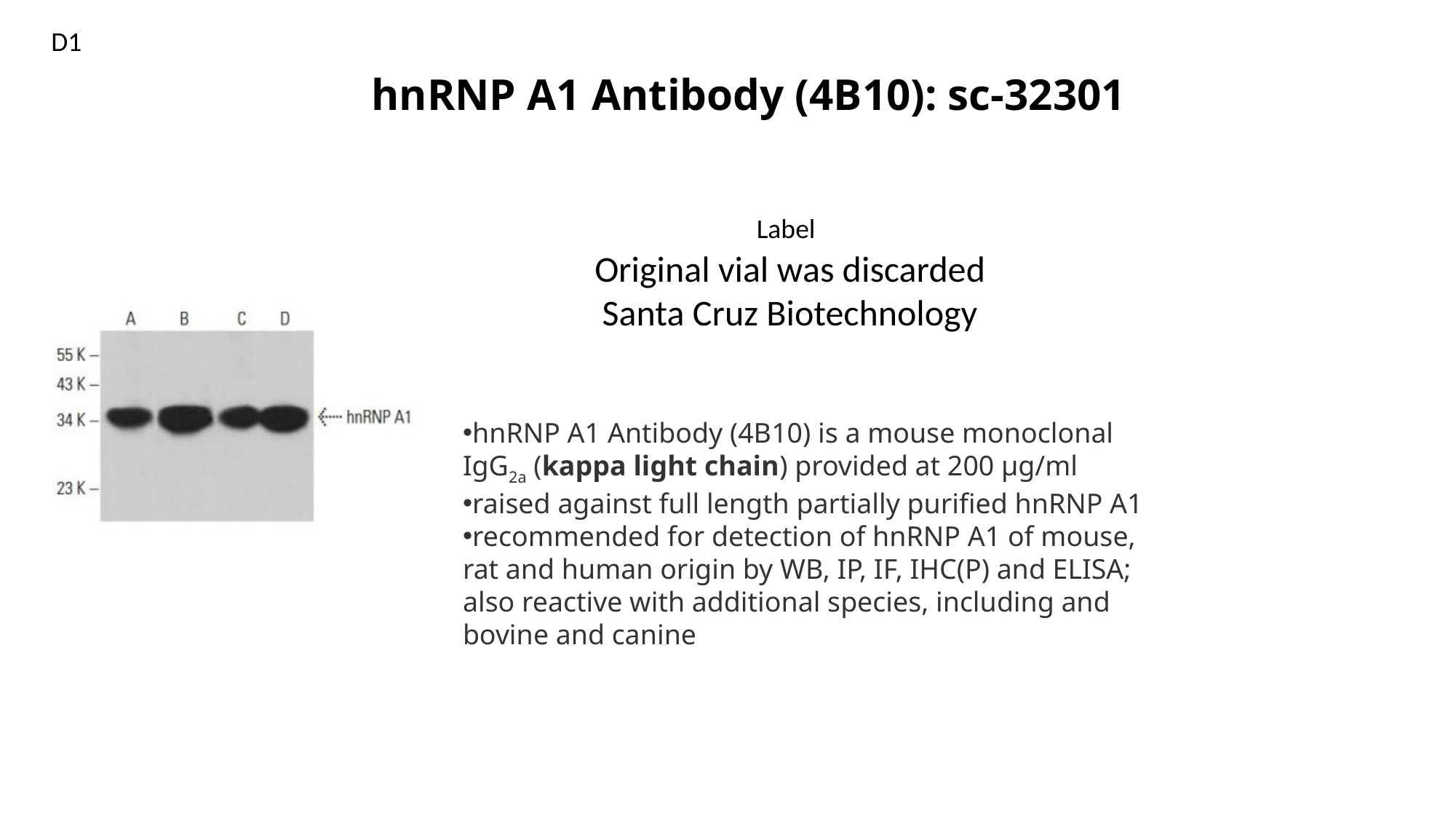

D1
hnRNP A1 Antibody (4B10): sc-32301
Label
Original vial was discarded
Santa Cruz Biotechnology
hnRNP A1 Antibody (4B10) is a mouse monoclonal IgG2a (kappa light chain) provided at 200 µg/ml
raised against full length partially purified hnRNP A1
recommended for detection of hnRNP A1 of mouse, rat and human origin by WB, IP, IF, IHC(P) and ELISA; also reactive with additional species, including and bovine and canine

## Slide 12
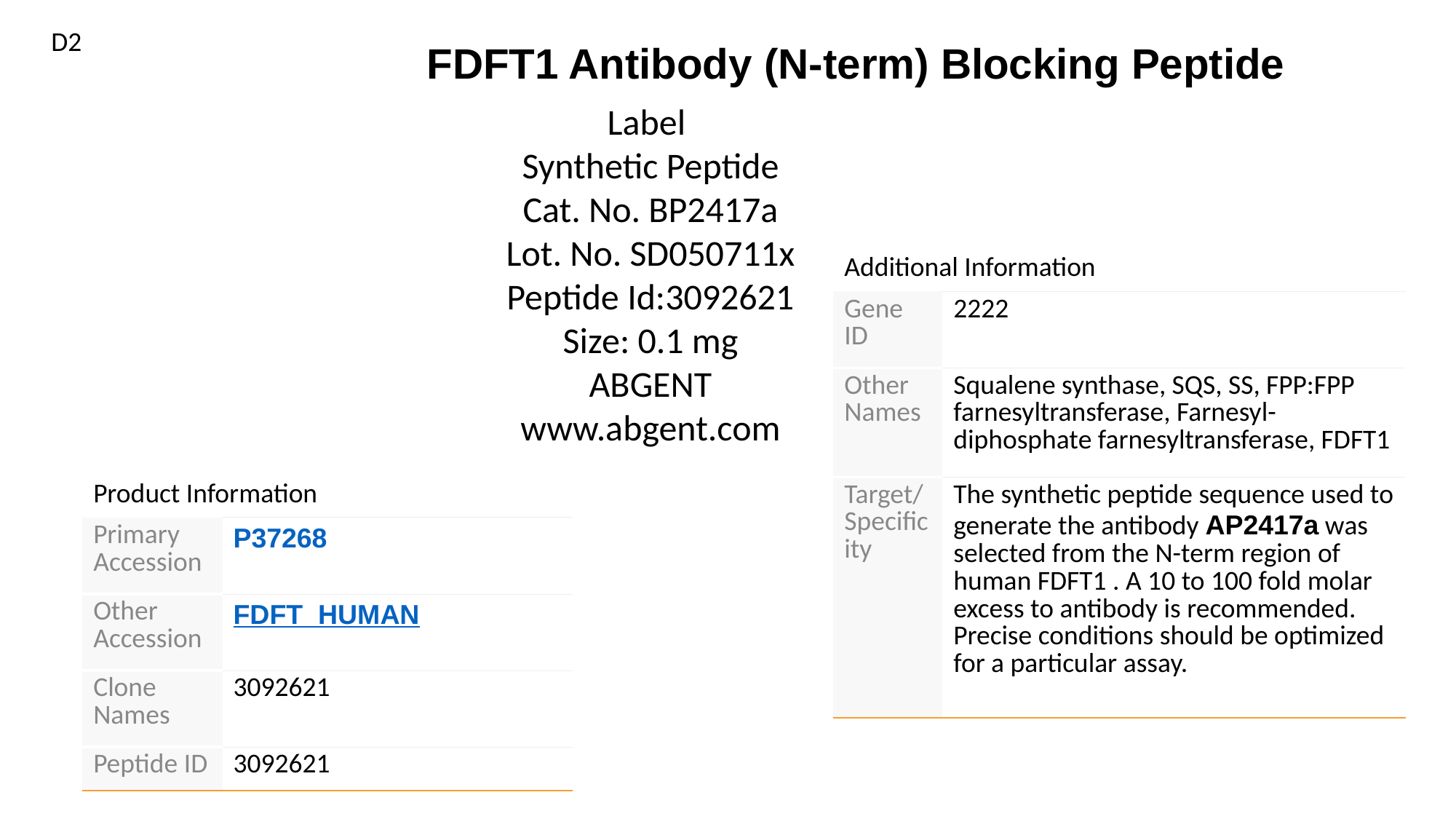

D2
FDFT1 Antibody (N-term) Blocking Peptide
Label
Synthetic Peptide
Cat. No. BP2417a
Lot. No. SD050711x
Peptide Id:3092621
Size: 0.1 mg
ABGENT
www.abgent.com
| Additional Information | |
| --- | --- |
| Gene ID | 2222 |
| Other Names | Squalene synthase, SQS, SS, FPP:FPP farnesyltransferase, Farnesyl-diphosphate farnesyltransferase, FDFT1 |
| Target/Specificity | The synthetic peptide sequence used to generate the antibody AP2417a was selected from the N-term region of human FDFT1 . A 10 to 100 fold molar excess to antibody is recommended. Precise conditions should be optimized for a particular assay. |
| Product Information | |
| --- | --- |
| Primary Accession | P37268 |
| Other Accession | FDFT\_HUMAN |
| Clone Names | 3092621 |
| Peptide ID | 3092621 |

## Slide 13
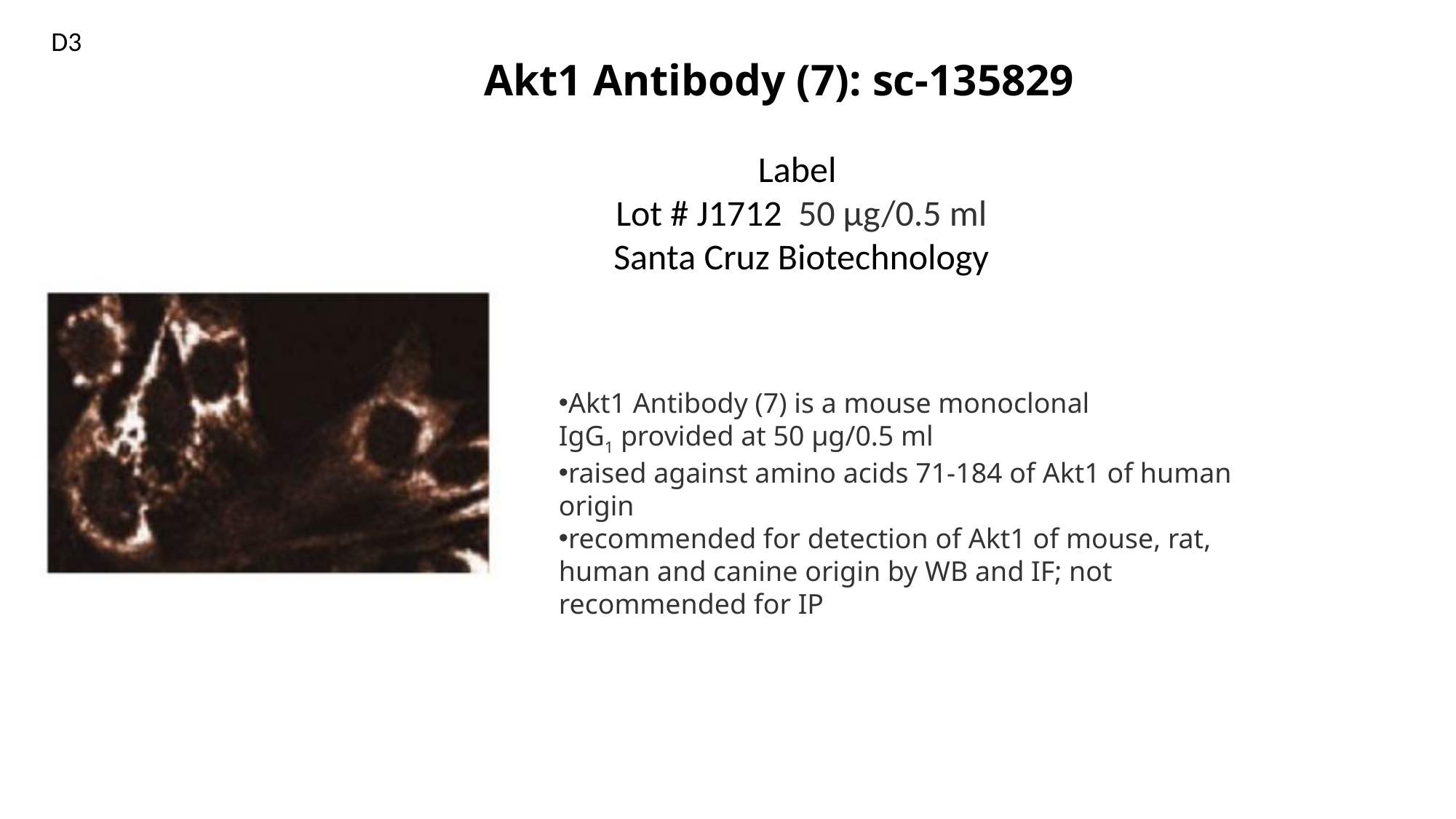

D3
Akt1 Antibody (7): sc-135829
Label
Lot # J1712 50 µg/0.5 ml
Santa Cruz Biotechnology
Akt1 Antibody (7) is a mouse monoclonal IgG1 provided at 50 µg/0.5 ml
raised against amino acids 71-184 of Akt1 of human origin
recommended for detection of Akt1 of mouse, rat, human and canine origin by WB and IF; not recommended for IP

## Slide 14
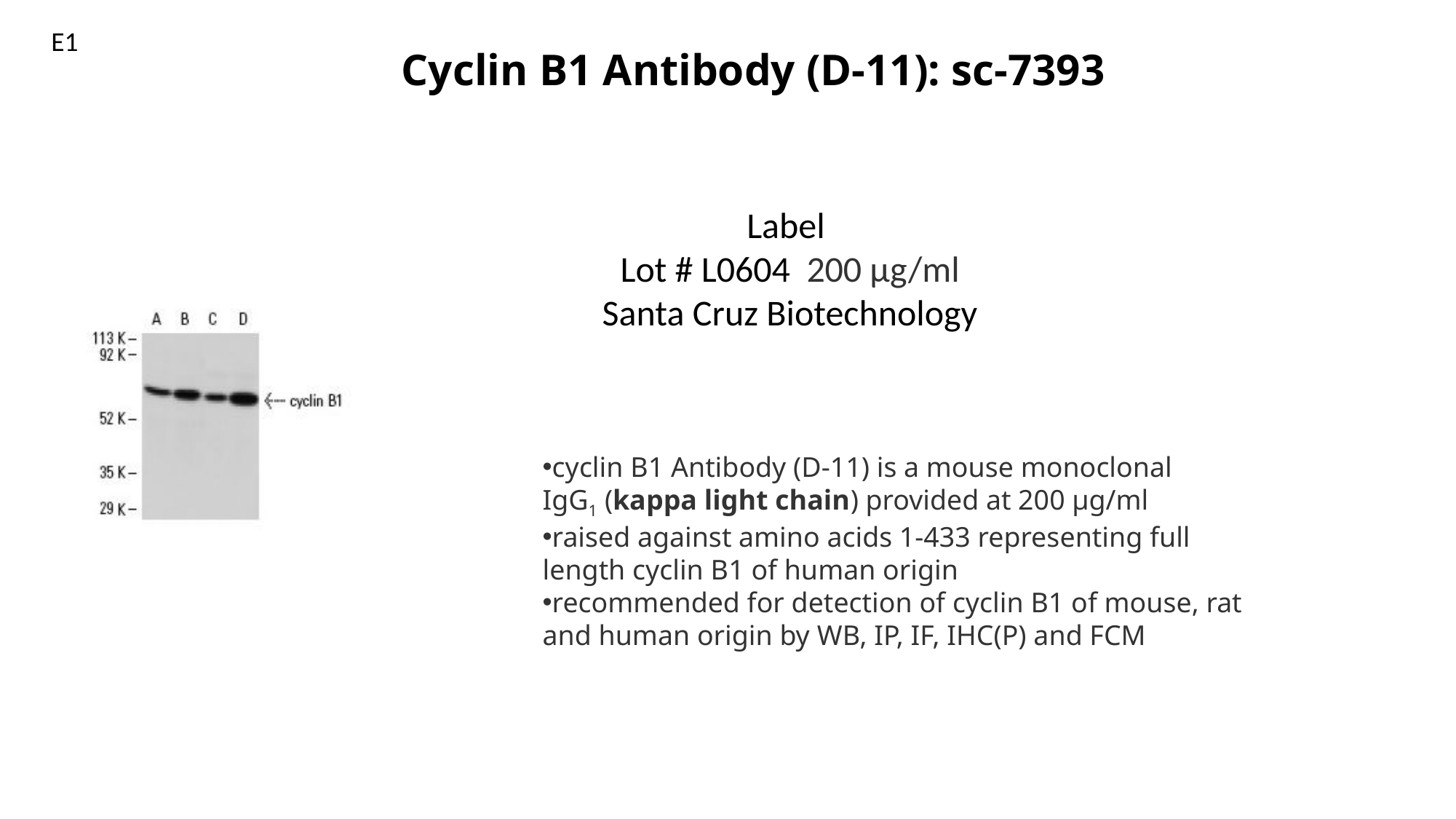

E1
Cyclin B1 Antibody (D-11): sc-7393
Label
Lot # L0604 200 µg/ml
Santa Cruz Biotechnology
cyclin B1 Antibody (D-11) is a mouse monoclonal IgG1 (kappa light chain) provided at 200 µg/ml
raised against amino acids 1-433 representing full length cyclin B1 of human origin
recommended for detection of cyclin B1 of mouse, rat and human origin by WB, IP, IF, IHC(P) and FCM

## Slide 15
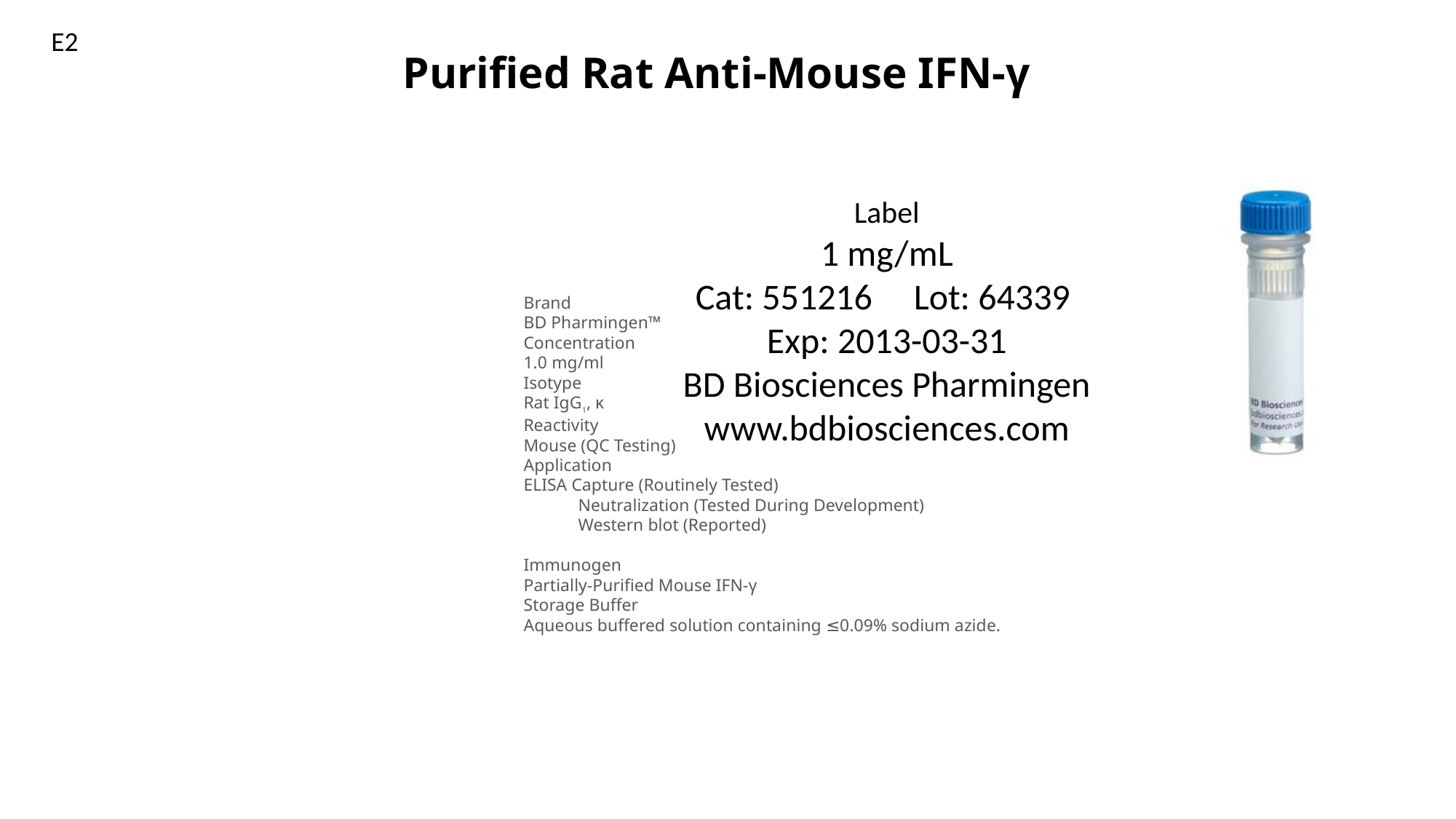

E2
Purified Rat Anti-Mouse IFN-γ
Label
1 mg/mL
Cat: 551216	Lot: 64339
Exp: 2013-03-31
BD Biosciences Pharmingen
www.bdbiosciences.com
Brand
BD Pharmingen™
Concentration
1.0 mg/ml
Isotype
Rat IgG1, κ
Reactivity
Mouse (QC Testing)
Application
ELISA Capture (Routinely Tested) 
Neutralization (Tested During Development) 
Western blot (Reported)
Immunogen
Partially-Purified Mouse IFN-γ
Storage Buffer
Aqueous buffered solution containing ≤0.09% sodium azide.

## Slide 16
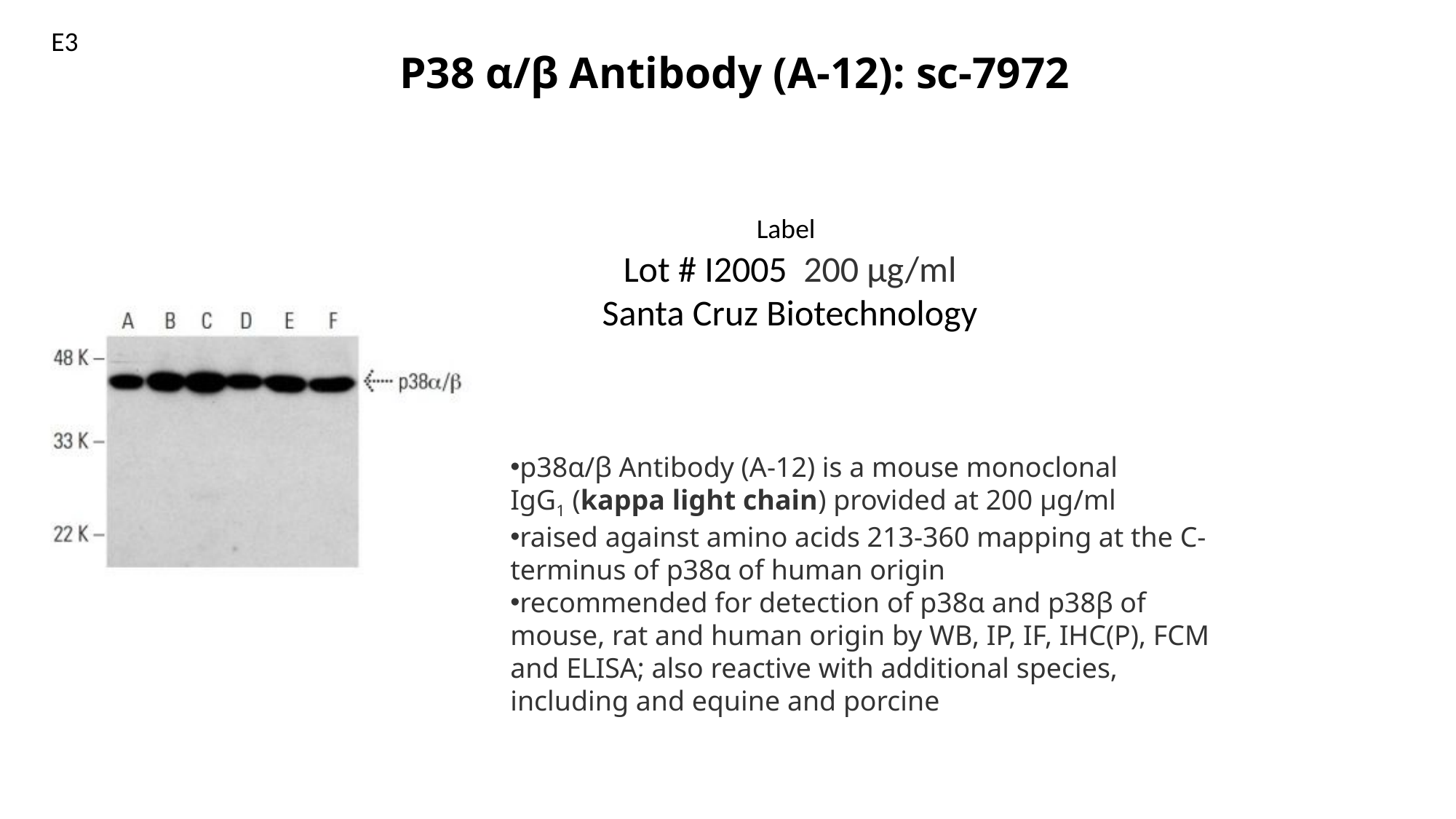

E3
P38 α/β Antibody (A-12): sc-7972
Label
Lot # I2005 200 µg/ml
Santa Cruz Biotechnology
p38α/β Antibody (A-12) is a mouse monoclonal IgG1 (kappa light chain) provided at 200 µg/ml
raised against amino acids 213-360 mapping at the C-terminus of p38α of human origin
recommended for detection of p38α and p38β of mouse, rat and human origin by WB, IP, IF, IHC(P), FCM and ELISA; also reactive with additional species, including and equine and porcine

## Slide 17
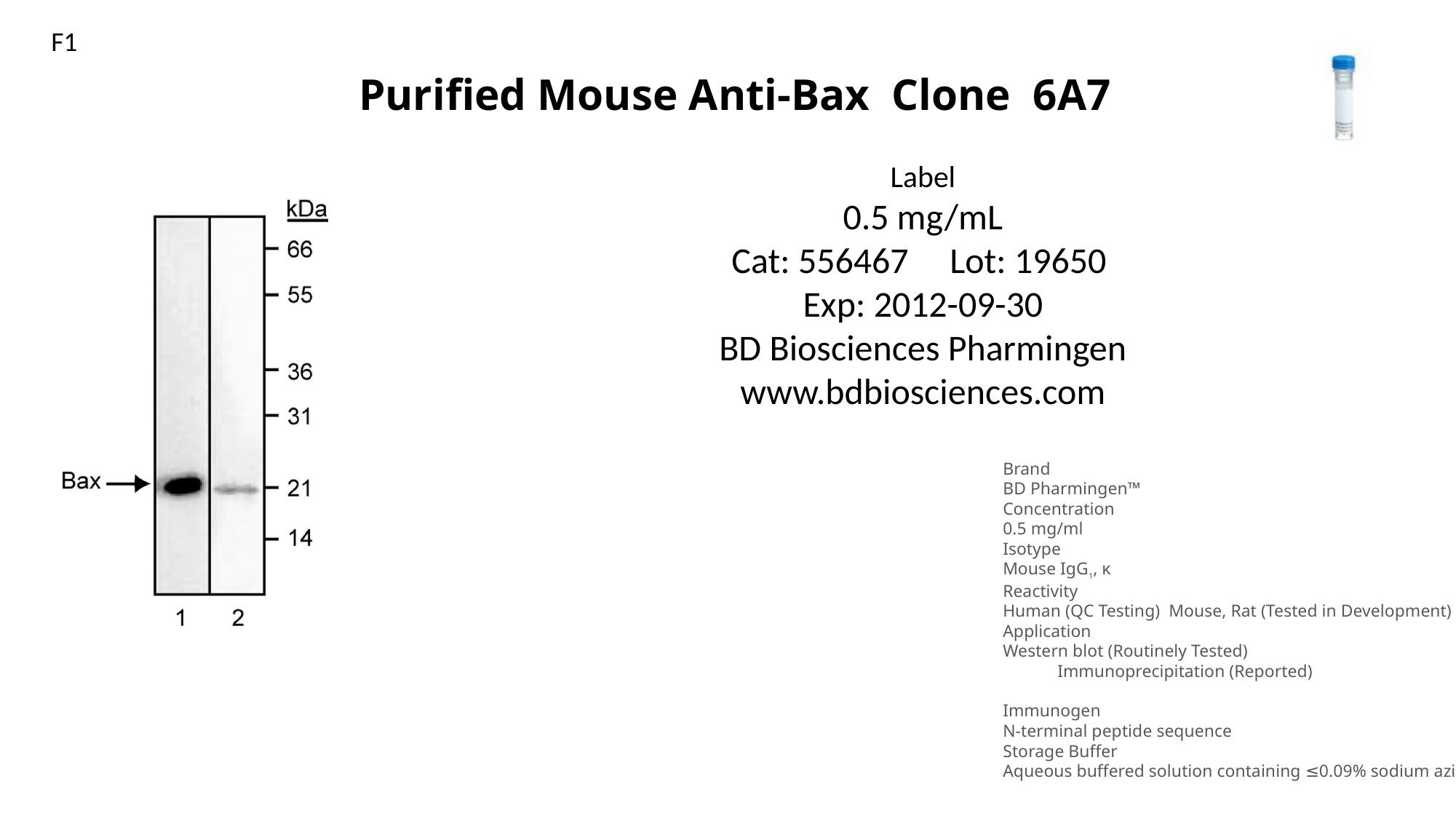

F1
Purified Mouse Anti-Bax  Clone  6A7
Label
0.5 mg/mL
Cat: 556467	Lot: 19650
Exp: 2012-09-30
BD Biosciences Pharmingen
www.bdbiosciences.com
Brand
BD Pharmingen™
Concentration
0.5 mg/ml
Isotype
Mouse IgG1, κ
Reactivity
Human (QC Testing)  Mouse, Rat (Tested in Development)
Application
Western blot (Routinely Tested) 
Immunoprecipitation (Reported)
Immunogen
N-terminal peptide sequence
Storage Buffer
Aqueous buffered solution containing ≤0.09% sodium azide.

## Slide 18
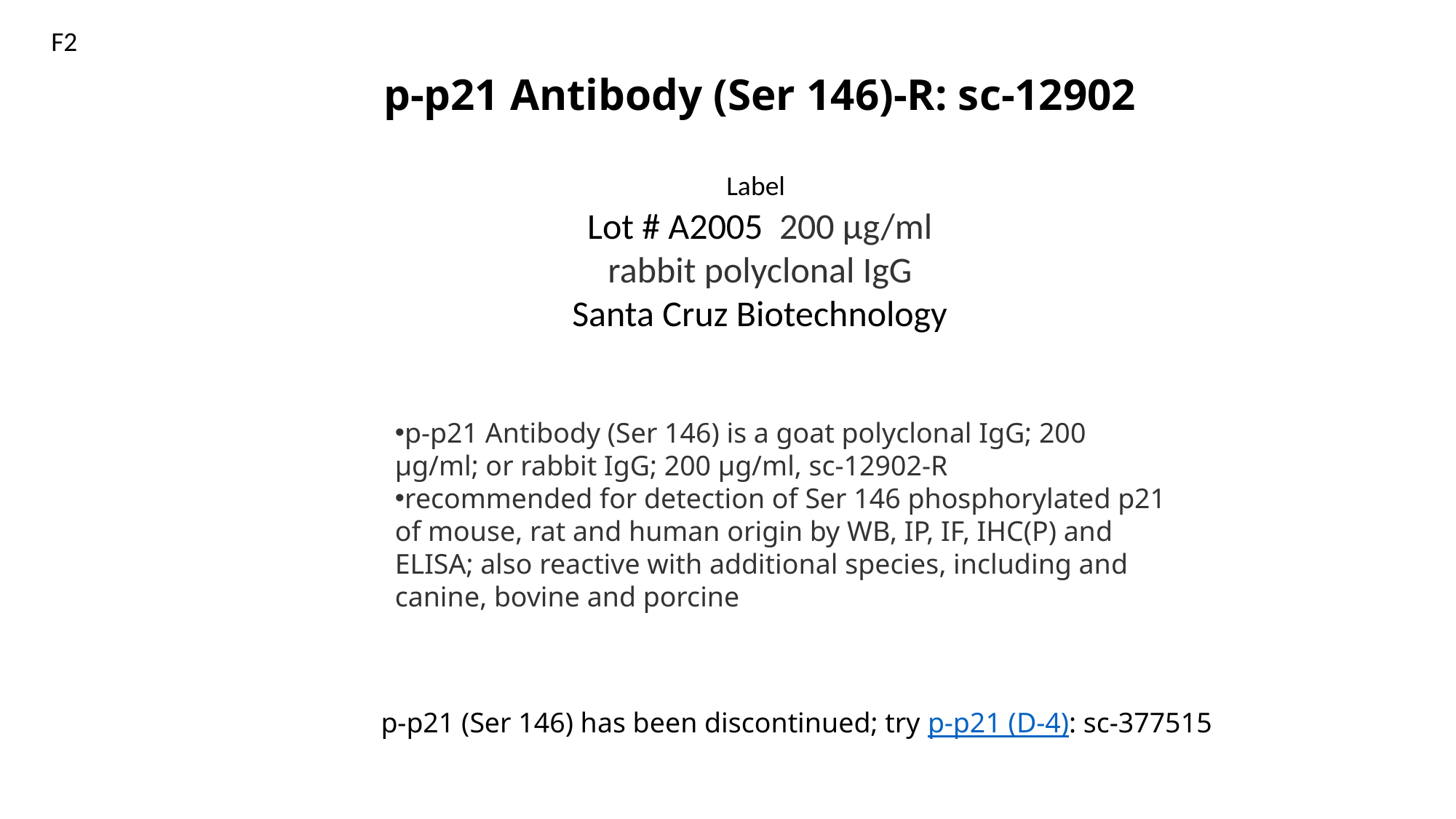

F2
p-p21 Antibody (Ser 146)-R: sc-12902
Label
Lot # A2005 200 µg/ml
rabbit polyclonal IgG
Santa Cruz Biotechnology
p-p21 Antibody (Ser 146) is a goat polyclonal IgG; 200 µg/ml; or rabbit IgG; 200 µg/ml, sc-12902-R
recommended for detection of Ser 146 phosphorylated p21 of mouse, rat and human origin by WB, IP, IF, IHC(P) and ELISA; also reactive with additional species, including and canine, bovine and porcine
p-p21 (Ser 146) has been discontinued; try p-p21 (D-4): sc-377515

## Slide 19
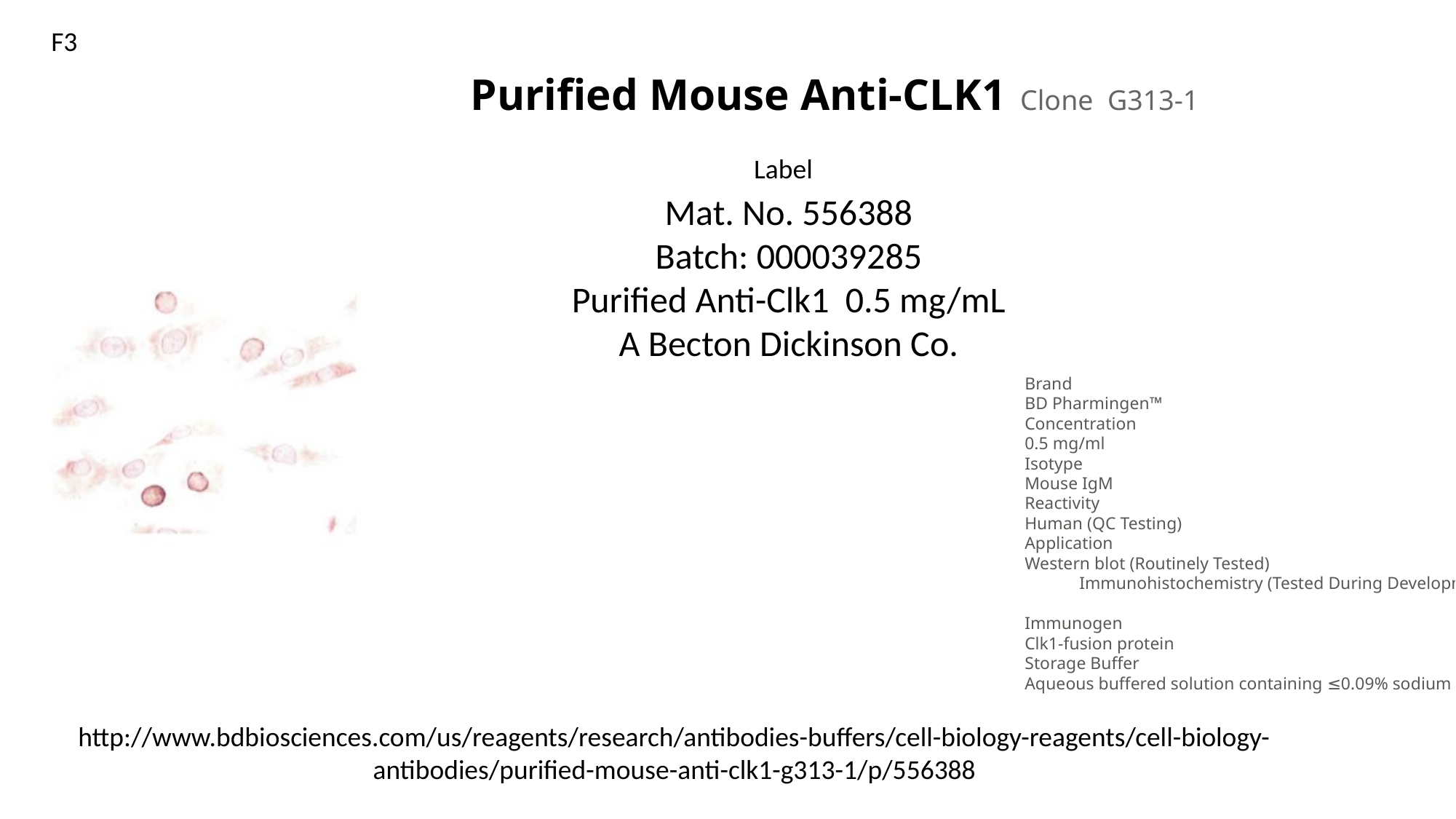

F3
Purified Mouse Anti-CLK1  Clone  G313-1
Label
Mat. No. 556388
Batch: 000039285
Purified Anti-Clk1 0.5 mg/mL
A Becton Dickinson Co.
Brand
BD Pharmingen™
Concentration
0.5 mg/ml
Isotype
Mouse IgM
Reactivity
Human (QC Testing)
Application
Western blot (Routinely Tested) 
Immunohistochemistry (Tested During Development)
Immunogen
Clk1-fusion protein
Storage Buffer
Aqueous buffered solution containing ≤0.09% sodium azide.
http://www.bdbiosciences.com/us/reagents/research/antibodies-buffers/cell-biology-reagents/cell-biology-antibodies/purified-mouse-anti-clk1-g313-1/p/556388

## Slide 20
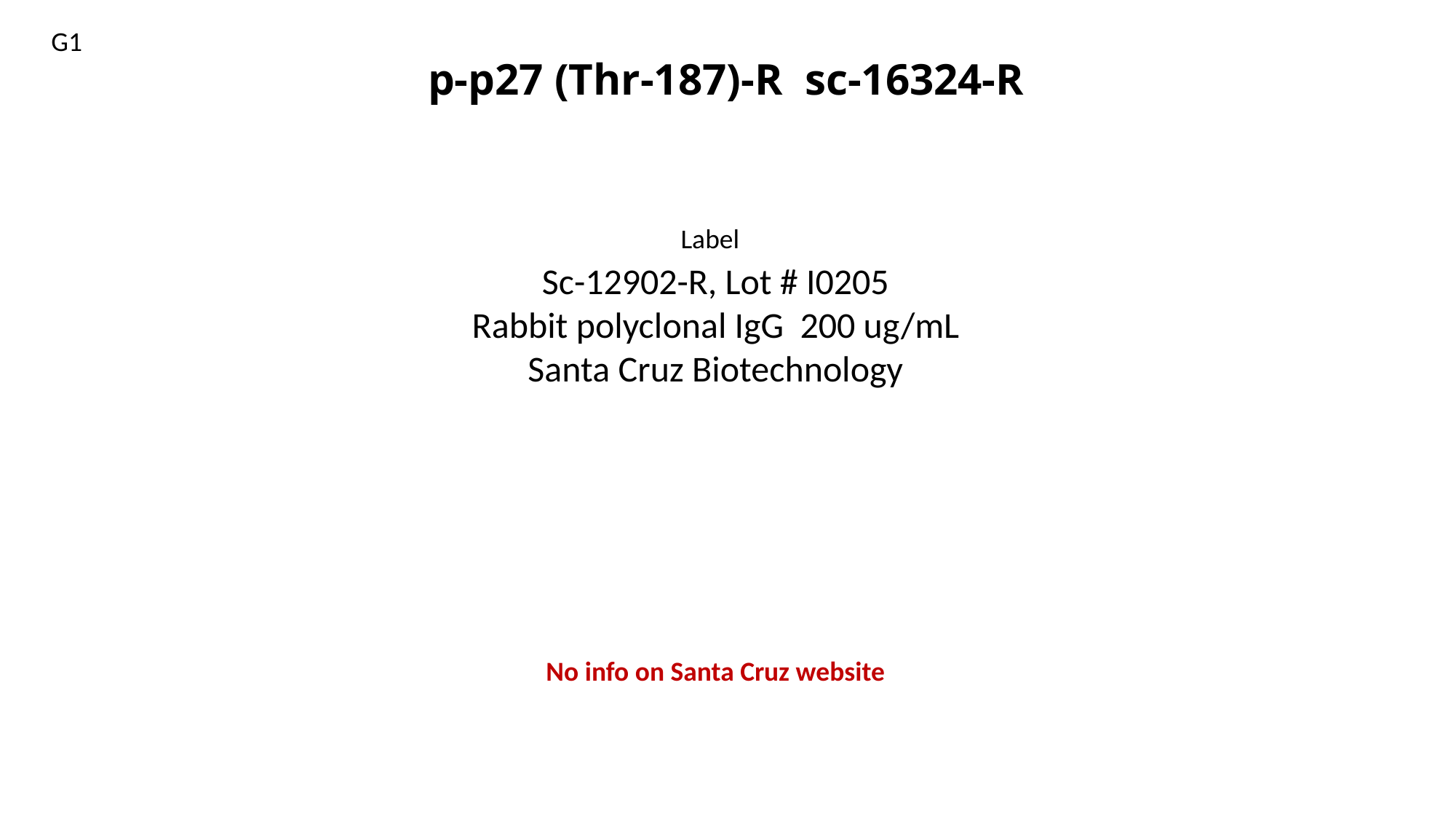

G1
p-p27 (Thr-187)-R sc-16324-R
Label
Sc-12902-R, Lot # I0205
Rabbit polyclonal IgG 200 ug/mL
Santa Cruz Biotechnology
No info on Santa Cruz website

## Slide 21
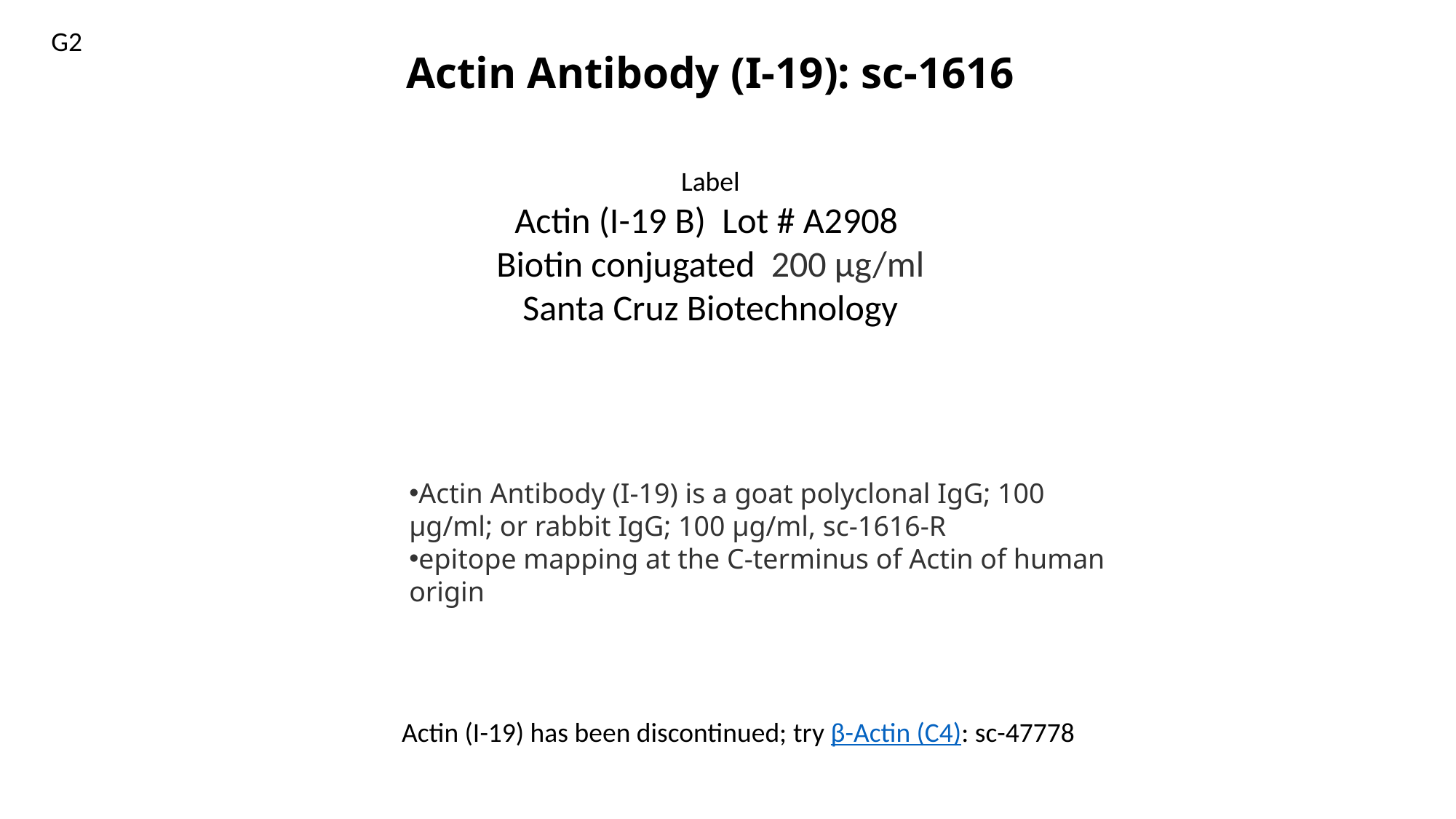

G2
Actin Antibody (I-19): sc-1616
Label
Actin (I-19 B) Lot # A2908
Biotin conjugated 200 µg/ml
Santa Cruz Biotechnology
Actin Antibody (I-19) is a goat polyclonal IgG; 100 µg/ml; or rabbit IgG; 100 µg/ml, sc-1616-R
epitope mapping at the C-terminus of Actin of human origin
Actin (I-19) has been discontinued; try β-Actin (C4): sc-47778

## Slide 22
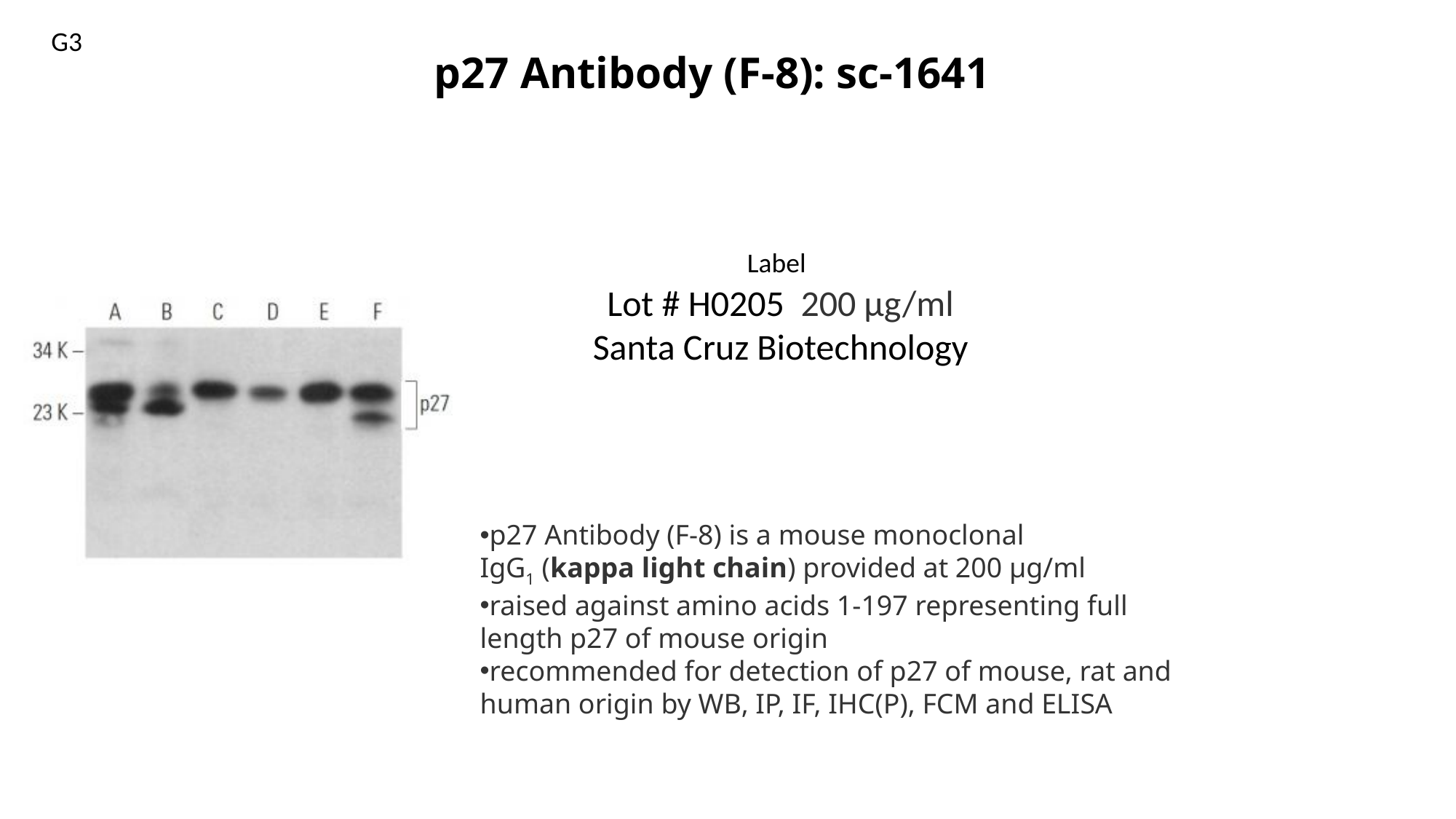

G3
p27 Antibody (F-8): sc-1641
Label
Lot # H0205 200 µg/ml
Santa Cruz Biotechnology
p27 Antibody (F-8) is a mouse monoclonal IgG1 (kappa light chain) provided at 200 µg/ml
raised against amino acids 1-197 representing full length p27 of mouse origin
recommended for detection of p27 of mouse, rat and human origin by WB, IP, IF, IHC(P), FCM and ELISA

## Slide 23
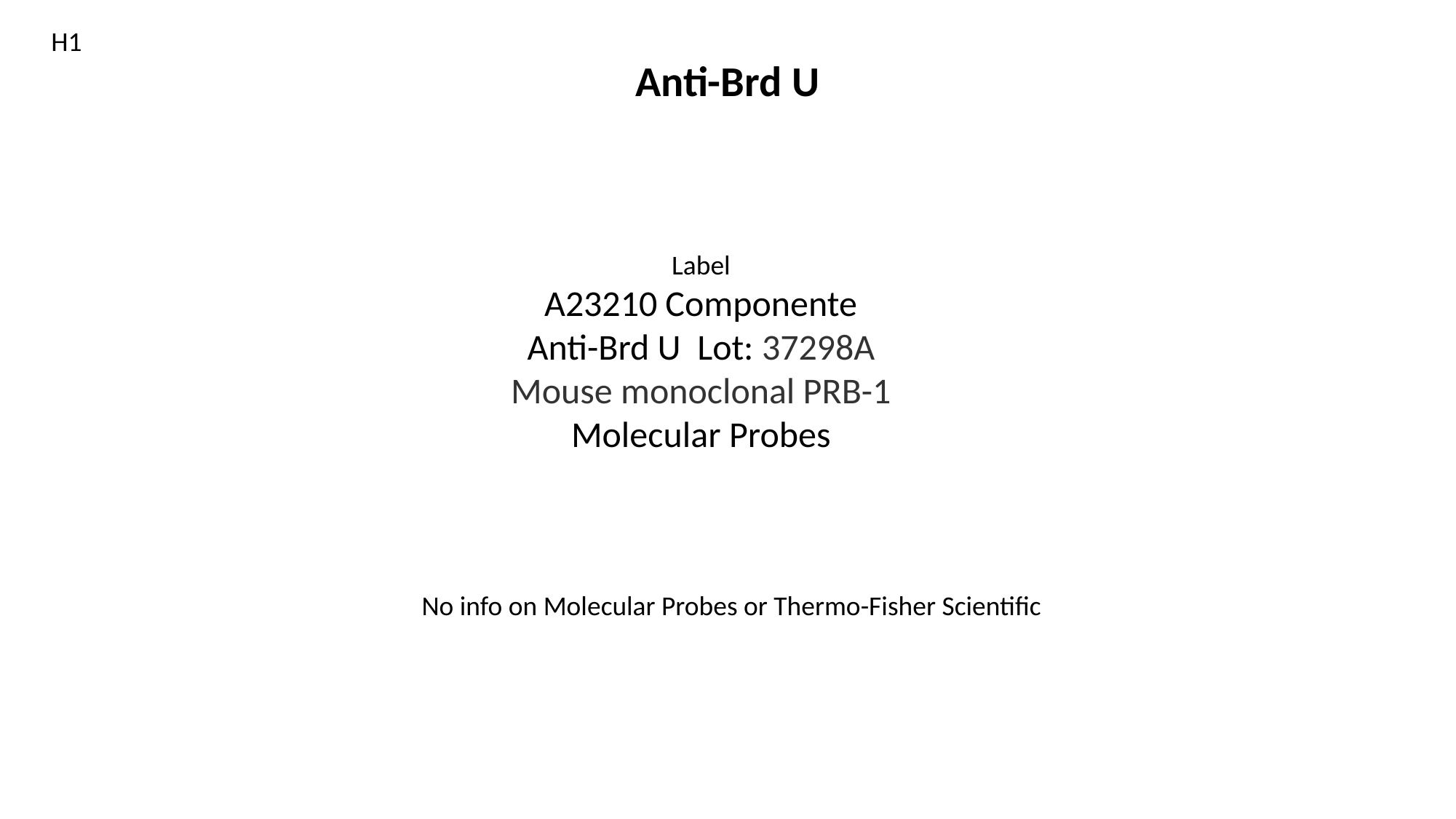

H1
Anti-Brd U
Label
A23210 Componente
Anti-Brd U Lot: 37298A
Mouse monoclonal PRB-1
Molecular Probes
No info on Molecular Probes or Thermo-Fisher Scientific

## Slide 24
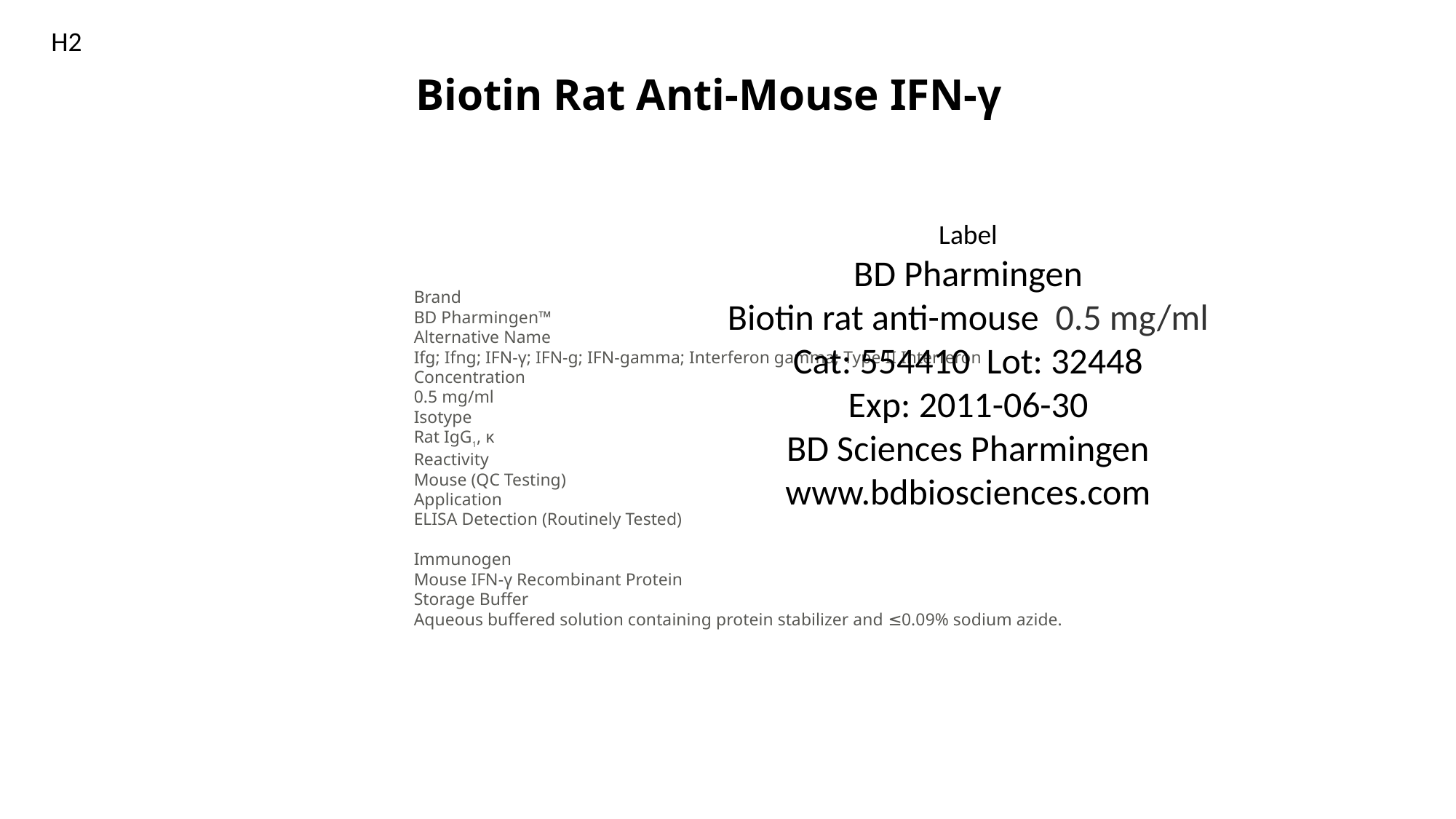

H2
Biotin Rat Anti-Mouse IFN-γ
Label
BD Pharmingen
Biotin rat anti-mouse 0.5 mg/ml
Cat: 554410 Lot: 32448
Exp: 2011-06-30
BD Sciences Pharmingen
www.bdbiosciences.com
Brand
BD Pharmingen™
Alternative Name
Ifg; Ifng; IFN-γ; IFN-g; IFN-gamma; Interferon gamma; Type II Interferon
Concentration
0.5 mg/ml
Isotype
Rat IgG1, κ
Reactivity
Mouse (QC Testing)
Application
ELISA Detection (Routinely Tested)
Immunogen
Mouse IFN-γ Recombinant Protein
Storage Buffer
Aqueous buffered solution containing protein stabilizer and ≤0.09% sodium azide.

## Slide 25
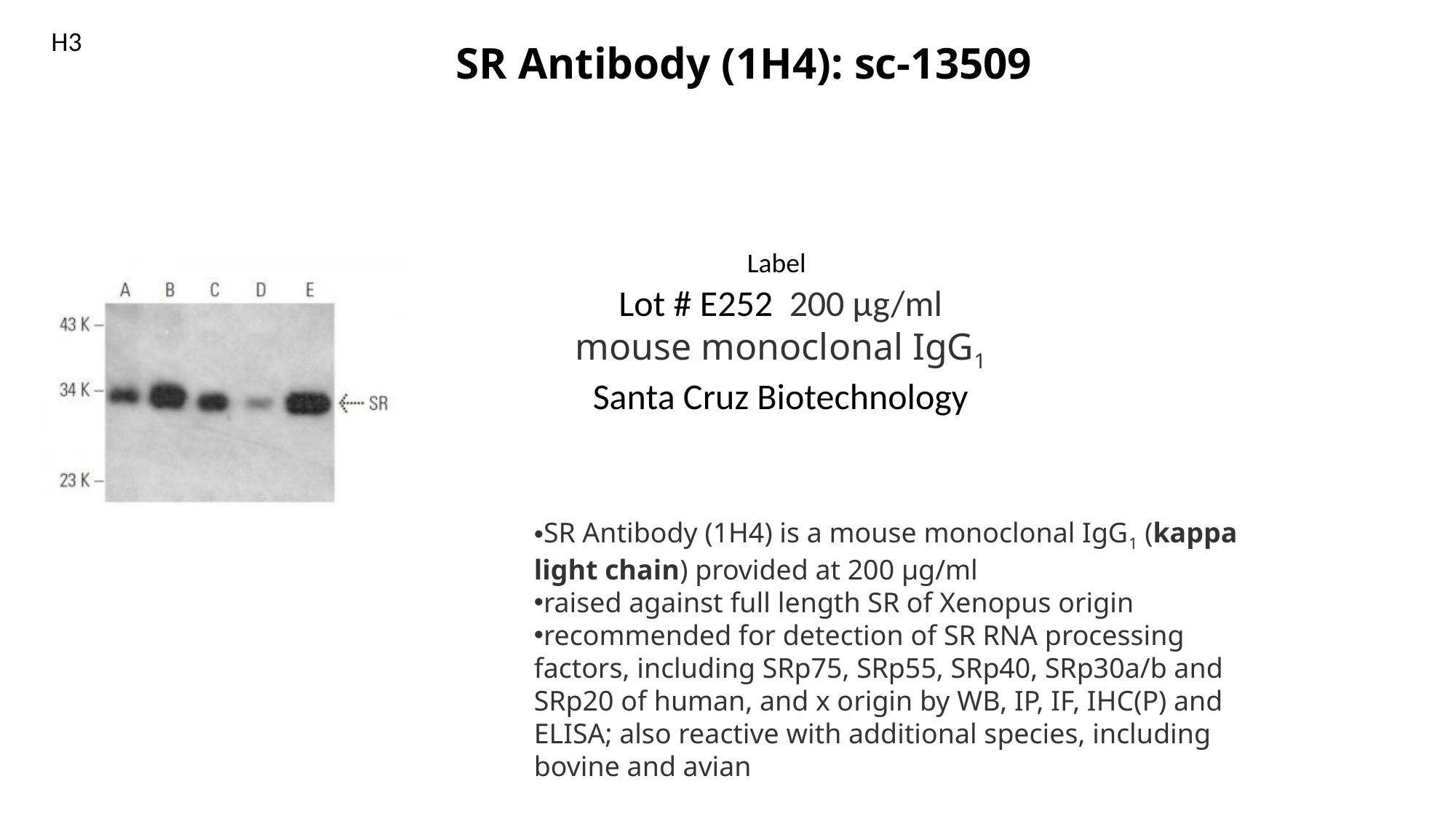

H3
SR Antibody (1H4): sc-13509
Label
Lot # E252 200 µg/ml
mouse monoclonal IgG1
Santa Cruz Biotechnology
SR Antibody (1H4) is a mouse monoclonal IgG1 (kappa light chain) provided at 200 µg/ml
raised against full length SR of Xenopus origin
recommended for detection of SR RNA processing factors, including SRp75, SRp55, SRp40, SRp30a/b and SRp20 of human, and x origin by WB, IP, IF, IHC(P) and ELISA; also reactive with additional species, including bovine and avian
